# Effectiveness of a Virtual Reality Serious Video Game (The Secret Trail of Moon) for Emotional Regulation in Children With Attention-Deficit/Hyperactivity Disorder: Randomized Clinical Trial

**DOI:** 10.2196/59124

**Published:** 2025-01-08

**Authors:** Marina Martin-Moratinos, Marcos Bella-Fernández, María Rodrigo-Yanguas, Carlos González-Tardón, Chao Li, Ping Wang, Ana Royuela, Pilar Lopez-Garcia, Hilario Blasco-Fontecilla

**Affiliations:** 1 Department of Psychiatry Health Research Institute of the Puerta de Hierro Majadahonda-Segovia de Arana University Hospital (IDIPHISA) Puerta de Hierro Majadahonda University Hospital Madrid Spain; 2 Faculty of Medicine Autonoma University of Madrid Madrid Spain; 3 Faculty of Psychology Autonoma University of Madrid Madrid Spain; 4 Department of Psychology Pontifical University of Comillas Madrid Spain; 5 Faculty of Health International Business University Madrid Spain; 6 Department of Video Games Design and Technology University Madrid Spain; 7 Sichuan Provincial Center for Mental Health University of Electronic Science and Technology of China Chengdu China; 8 Clinical Biostatistics Unit Puerta de Hierro Majadahonda University Hospital Madrid Spain; 9 Consortium for Biomedical Research Network in Epidemiology and Public Health Madrid Spain; 10 Spain Biomedical Research Networking Center for Mental Health Network Madrid Spain; 11 Health Sciences and School of Doctoral Programs International University of La Rioja Logroño Spain; 12 Emooti Institute for Mental Health and Wellness Madrid Spain

**Keywords:** attention-deficit/hyperactivity disorder, ADHD, emotional regulation, serious video games, virtual reality, cognitive training, music, chess

## Abstract

**Background:**

Difficulties in emotional regulation are often observed in children and adolescents with attention-deficit/hyperactivity disorder (ADHD). Innovative complementary treatments, such as video games and virtual reality, have become increasingly appealing to patients. *The Secret Trail of Moon* (MOON) is a serious video game developed by a multidisciplinary team featuring cognitive training exercises. In this second randomized clinical trial, we evaluated the impact of a 20-session treatment with MOON on emotional regulation, as measured by the Strengths and Difficulties Questionnaire.

**Objective:**

We hypothesize that patients with ADHD using MOON will show improvements in (1) emotional regulation, (2) core ADHD symptoms, (3) cognitive functioning, and (4) academic performance, compared to a control group; additionally, we anticipate that (5) changing the platform (from face-to-face using virtual reality to the web) will not affect emotional regulation scores; and (6) the video game will not cause any clinically significant side effects.

**Methods:**

This was a prospective, unicentric, randomized, unblinded, pre- and postintervention study with block-randomized sequence masking. Participants included individuals aged between 7 and 18 years who had a clinical diagnosis of ADHD and were receiving pharmacological treatment. They were randomized into 2 groups using an electronic case report form: the MOON group, receiving standard pharmacological treatment plus personalized cognitive training via a serious video game, and the control group, receiving standard pharmacological treatment. We provided both the groups with psychoeducational support on ADHD. Analysis was conducted using the Student 2-tailed *t* test and 2-factor ANOVA. An independent monitor supervised the study.

**Results:**

A total of 76 patients with ADHD participated in the trial, with an equal randomization (MOON: n=38, 50% and control: n=38, 50%) and a total dropout rate of 7. The primary hypothesis, a 3- or 4-point reduction in the global Strengths and Difficulties Questionnaire score, was not met. However, significant improvements were observed in material organization (*P*=.03), working memory (*P*=.04), and inhibition (*P*=.05), particularly among patients more engaged with the MOON treatment.

**Conclusions:**

Serious video games, when integrated into a multimodal treatment plan, can enhance outcomes for symptoms associated with ADHD.

**Trial Registration:**

ClinicalTrials.gov NCT06006871; https://clinicaltrials.gov/study/NCT06006871

**International Registered Report Identifier (IRRID):**

RR2-10.2196/53191

## Introduction

Attention-deficit/hyperactivity disorder (ADHD) is characterized by a persistent pattern of inattention, hyperactivity, and impulsivity, affecting approximately 5% of children and adolescents worldwide [1,2]. These symptoms significantly impair daily functioning across social, academic, cognitive, behavioral, and family domains. In addition to the core symptoms, emotional problems and poor self-regulation are increasingly seen as central features of ADHD [3-5]. Emotional regulation allows modifying the emotional state by promoting adaptive behaviors to achieve a goal [6]. Children and adolescents with ADHD often struggle with emotional regulation, exhibiting low frustration tolerance, irritability, and emotional lability. A recent meta-analysis highlighted difficulties associated with ADHD in emotional lability (*d*=0.95), emotional regulation (*d*=0.80), empathy (*d*=0.68), and emotion recognition or understanding (*d*=0.64), with differences associated with gender, age, and the existence of executive dysfunction or behavioral problems [7]. Internalizing and externalizing problems are common among individuals with ADHD [8]. In fact, comorbidity is highly prevalent in ADHD with internalizing disorders such as anxiety or depression as well as externalizing disorders such as conduct disorder and oppositional defiant disorder [9]. The camouflaging of symptoms [10] often leads to underdiagnosis and treatment delays, resulting in misdiagnosis, higher rates of accidents, use of public services, increased likelihood of substance use, delinquency, and even suicide attempts [11-13].

Multimodal treatment for ADHD includes pharmacological treatment, psychological treatment, and psychoeducation for parents and teachers. While multimodal treatment is the most effective for ADHD, it can sometimes be insufficient, requiring complementary treatments [14]. Pharmacological treatment is the treatment of choice in children and adolescents with severe ADHD [15]. Stimulant treatment reduced behaviors linked to emotional instability, such as substance use, suicide rate, or criminal behavior [5,13]. Behavioral therapy, particularly focused on problem-solving and social skills training, is the recommended form of psychotherapy for ADHD [16,17]. Nevertheless, some areas, such as executive function, emotional regulation, and core ADHD symptoms, may show limited improvement through multimodal treatment alone. Motivation, especially intrinsic motivation, and responsiveness to insufficient reward plays a critical role in ADHD [18]. Individuals with ADHD often seek higher levels of immediate reward, which can lead to neglect tasks, dropped jobs, or incomplete treatments. Complementary treatments have emerged that are more attractive to patients, such as the use of games or video games or new technologies such as virtual reality (VR) [19,20]. Children with ADHD who engaged in working memory training incorporating game elements demonstrated longer training durations, completed more sequences with fewer errors, and achieved higher scores compared to those who underwent standard cognitive training [21]. When motivated, children with ADHD perform comparably to typically developing children on most cognitive tasks and may even outperform them on engaging computer-based tasks, despite the need for sustained attention [18].

In this prospective, unicentric, unblinded, pre- and postintervention randomized clinical trial (NCT06006871), we investigated the efficacy of *The Secret Trail of Moon* (MOON) for improving emotional regulation in people with ADHD aged between 7 and 18 years. MOON is a serious video game designed for health purposes, developed by a multidisciplinary team; the environment is composed of a forest with the intention of immersing the player through the sensation of presence that VR facilitates [22,23]. VR favors a lower perception of the real physical environment [22] and, therefore, a lower distraction by stimulation, a key aspect for a treatment for individuals with ADHD [15,24]. The video game experience can be immersive and exciting. A very low level of arousal can lead to boredom and reduced attention to the game. Conversely, if the player experiences too much arousal, it can also divert attention [22]. Music can help modulate these emotional states besides facilitating flow and immersion while playing video games [25]. Particularly in ADHD, music can be beneficial in reducing environmental distraction, especially in monotonous tasks [25,26]. Elements of music therapy have been both passively and interactively incorporated into this new version of the game [25,27]. Finally, an additional rhythm-based game mechanic (active music therapy) could not be added in the clinical trial due to its nonapproval by the ethics committee. The video game integrates bright but calm colors in a cartoon style, friendly characters that accompany the patient in the process, and varied cognitive training games based on neuropsychological tests: (1) Smasher (sustained attention and inhibitory control); (2) Teka Teki (planning); and (3) Kuburi (visuospatial capacity), Chess (reasoning), and Enigma (working memory). The games increase in difficulty level according to patient performance [28]. A previous clinical trial (NCT04355065) showed promising results in emotional intelligence, emotional regulation, and performance in the school context in both self-reports and parent reports [29]. Contrary to our expectations, no significant differences were observed in the improvement of executive functions, as measured by the Behavior Rating Inventory Executive Function, version 2 (BRIEF-2), between the video game group and the control group. Compared to the previous clinical trial, this time the study design is simplified into 2 branches: experimental (play MOON in the hospital and at home during 20 sessions) versus control (do not play MOON and telephone follow-up to parents). Both groups have stable pharmacological treatment and psychoeducation to parents. We anticipated a 3- to 4-point reduction in the global Strengths and Difficulties Questionnaire (SDQ) score between the baseline assessment (day 0) and the final assessment (day 90), reflecting an improvement in emotional regulation. As secondary measures, we propose 6 additional hypotheses:

- Hypothesis 1—ADHD patients using the MOON video game improve emotional regulation compared to the control group
- Hypothesis 2—patients with ADHD using the MOON video game improve core ADHD symptoms compared with the control group
- Hypothesis 3—patients with ADHD using MOON improve their cognitive functioning compared to the control group
- Hypothesis 4–patients with ADHD using MOON improve in academic performance with respect to the control group
- Hypothesis 5—a change in platform (hospital-based, face-to-face VR vs home-based, web-based computer version) will not result in differences in emotional regulation outcomes
- Hypothesis 6—there is no clinically meaningful side effects associated with the video game

## Methods

### Study Design

Prospective, unicentric, randomized, unblinded, pre- and postintervention study with a masked randomization sequence by blocks (NCT06006871) [30]. Participants were randomized by electronic case report form (eCRF) in 2 groups: group 1 (MOON), which received standard pharmacological treatment combined with personalized cognitive training through a serious video game designed for patients with ADHD, along with psychoeducational support for parents; and group 2 (control), which received standard pharmacological treatment and psychoeducational support for parents, without the video game intervention. The study followed a parallel assignment model (MOON vs control) with a 1:1 allocation ratio.

No major changes were made to the methodology before the commencement of the clinical trial. In this randomized clinical trial, the study design and reporting adhered to the CONSORT 2010 guidelines, specifically following the CONSORT checklist for reporting information in randomized trials (Multimedia Appendix 1). The research questions were formulated using the Population, Intervention, Comparison, Outcomes and Study framework. The study protocol is published [30] and registered on ClinicalTrials.gov (NCT06006871) with International Registered Report Identifier (DERR1-10.2196/53191).

### Ethical Considerations

This study received approval from the Research Ethics Committee of the Puerta de Hierro University Hospital on December 14, 2022 (PI 106/22). Authorization was subsequently granted by the Spanish Agency of Medicines and Health Products on February 14, 2023 (1061/22/EC-R). The study was monitored by an independent monitor. All participants signed the informed consent form. Data were anonymized through the assignment of a clinical trial-specific code. The data were treated confidentially in accordance with the Organic Law 3/2018 of December 5 on the protection of personal data and guarantee of digital rights. This study complied with the standards of good clinical practice and the declaration of Helsinki. Participants were not compensated financially.

### Study Population

Children and adolescents aged between 7 and 18 years (mean age 12.68, SD 2.75 years) with a clinical diagnosis of ADHD according to the *Diagnostic and Statistical Manual of Mental Disorders, Fifth Edition*, were enrolled in the study. All participants had a clinical diagnosis of ADHD in any presentation and were receiving stable pharmacological treatment for ADHD. Patients were clinically stable, with a Clinical Global Impression (CGI) score between 3 and 6 before entering the clinical trial. Comorbidity was not an exclusion criterion in the study, except for those patients with suicide risk, lack of ability to follow verbal instructions, or motor difficulties that make it difficult to play a video game. Exclusion criteria also included participation in similar video games studies or the intention to initiate psychotherapeutic treatment during the 3-month study period. Medication was not altered during the study, unless adjustments were necessary for clinical reasons. A dropout rate of 15% was expected.

The randomized clinical trial had a total duration of 7 months, concluding on December 15, 2023. Recruitment took place between May and October 2023. The first evaluation was performed on May 9, 2023. The last evaluation was performed on October 31, 2023.

The intention-to-treat (ITT) population was defined as randomized participants who completed at least 10 sessions. The per-protocol (PP) population was defined as participants who completed 100% of the training. Only 32% (12/38) participants in the MOON group completed all 20 treatment sessions (PP), so we have not performed the analyses with this small sample size. In the ADHD population, adherence difficulties are common in long-term treatments; therefore, we planned to conduct the analyses on the premise of the ITT analysis. An additional analysis was also included to evaluate those participants who engaged with the intervention by completing 80% treatment (16 sessions in the MOON group or engagement for 8 weeks in the control group). Recognizing that the number of sessions could be a relevant covariate, we performed an analysis of covariance (ANCOVA), which was not initially included in the protocol. Only half of the parents (38/76, 50%; Multimedia Appendix 2) provided information on academic grades. Therefore this primary outcome (hypothesis 4) was excluded due to insufficient data.

### Procedure

All participants were recruited from the child and adolescent psychiatry outpatient clinics of Hospital Universitario Puerta de Hierro Majadahonda. The MOON group had 12 face-to-face visits, whereas the control group had only 2 presential visits: one at the preevaluation visit (D0) and another at the postevaluation visit (D90). The total duration of the research was 3 months for each participant. During the preinclusion phase, the principal investigator (HB-F) informed eligible patients about the procedure and research protocol. A total of 155 participants were assessed for enrollment. The study coordinator (MM-M) provided a detailed explanation of the research procedure and summoned the prerecruited participants. In total, 7.7% (12/155) of the patients did not meet the inclusion criteria (main reasons were clinical CGI, no medication, or age); 43.2% (67/155) of the patients declined to participate (the most common reason being lack of availability).

During the inclusion visit (D0), the research procedure was thoroughly explained, the informed consent form was signed, and the preevaluation was conducted (refer to the Study Outcomes section). Afterward, participants were randomly assigned to groups. Group randomization (MOON vs control) was performed using block randomization through the electronic eCRF REDCap (Research Electronic Data Capture; Vanderbilt University) with a 1:1 allocation ratio.

The intervention training (D1-D90) varied according to the assigned group. Parents in the control group received weekly telephone monitoring. For the MOON group, 20 sessions with the video game were scheduled: 10 sessions were conducted in the hospital with the researchers, and 10 sessions took place on the web at home, with participants using their computers under the researchers’ supervision (twice a week, adjusted according to the availability of the participants). Patients aged >12 years played MOON with VR, while those aged <12 years performed the video game sessions on a computer, following PlayStation guidelines. MM-M and MB-F assisted participants during their initial use of the video game, providing instructions, encouraging progress, and helping them manage any frustration caused by mistakes made while playing MOON. They also monitored for signs of motion sickness with VR, ensured proper use of the software, and addressed any potential frustration resulting from bugs in the video game. If a patient reported dizziness, even mild, they were instructed to remove the device and were given the option to decide whether or not to continue using it. All participants followed the same order pattern of the MOON games during 20 minutes of gameplay (refer to the study by Martin-Moratinos et al [30] for details).

In week 5 (D45), all participants’ parents receive a follow-up questionnaire with the main variable (SDQ). For the MOON group, both parents and children were trained on how to use the video game at home, using a flash drive. Parents and patients were additionally given an instruction manual. Monitoring was provided by telephone where the researchers checked the correct playability at home. The web-based sessions conducted were monitored by MM-M through the PlayFab data server (Microsoft Corporation). All participants had their personal username and password connected with PlayFab data server.

In the final visit (D90), a postevaluation was conducted using the same questionnaires, and participants were provided with feedback on the most relevant changes observed.

### Materials

The MOON video game was designed with the Unity (version 2020.3.7; Unity Technologies) software tool by a multidisciplinary team. Autodesk 3ds Max, Autodesk Maya, Adobe Photoshop, and PreSonus Studio 4 programs were used for the modeling of characters and landscape elements. In relation to the narrative and identity features [31,32], the player is immersed in a forest with archaic chess statues of an ancient civilization [29,31]. The raccoon Movi and the fox One will help the player to solve the puzzles (game mechanics). MOON is mainly composed of a menu screen and 5 different game mechanics focused on cognitive training.

To provide manipulation and control features [32], each player has their own user profile with saved progress. Each test is set in a closed environment with distinct esthetics and game mechanics. The manipulation of the environment and interactivity are carefully controlled to avoid distractions or dizziness in the VR setting [28]. For example, in Kuburi, blocks can be rotated and moved within the space, while in Chess, the player can shift positions to view the board from different perspectives. The mobility within environments is restricted based on the individual’s cognitive capacity. In cases where mobility could cause distraction—such as in tests of sustained attention or working memory, which are more challenging for individuals with ADHD—exploration is limited.

In all game mechanics, players begin at a base level (tutorial, level 1) to ensure they understand the task. The difficulty increases in a personalized manner based on performance, ensuring the game is neither too easy (and thus boring) nor too difficult (and thus frustrating). Emotional regulation maturity, specifically the ability to reappraise, can be trained through controlled exposure to negative emotional stimuli, helping players overcome levels and obstacles within the game [31]. Regarding reward and punishment features [32], the MOON video game uses a star system based on player performance (0 stars=poor performance, must repeat the level; 1 star=acceptable; 2 stars=good; and 3 stars=excellent). Player progress in each game mechanic is communicated through level advancements, performance parameters, and the number of stars earned [30].

In terms of music, each game mechanic features different tracks [25]. The music was specifically designed to align with cognitive abilities. For example, sustained attention, which requires more effort for individuals with ADHD [24], benefits from dynamic soundtracks. To prevent monotony and boredom, more epic, fast-paced music was composed for Smasher to maintain motivation through its rhythm. Feedback is not only visual but also auditory, with distinct sounds depending on user interactions. Different sounds were created to indicate level selection and hits or misses, as well as to signal conditions of victory (successfully completing a level) or defeat (repeating the level after earning 0 stars due to poor performance) [25]. In this clinical trial, the same version of the video game was proposed in 2 platforms: VR and computer (Table 1) [30].

**Table 1:** Hardware and software specifications categorized by The Secret Trail of Moon (MOON) gameplay on virtual reality (VR) or computer.

MOON	Patients’ target	Hardware	Software
VR	For children aged >12 years (following PlayStation guidelines) Only the first 10 sessions with on-site supervision at the hospital by researchers	PlayStation 4 Dev Kit and Test Kit PlayStation VR headset PlayStation camera Computer screen Controller: DualShock 4 Headphones with 3D stereo sound and noise cancelation	Gameplay: the player’s camera is the VR viewer
Computer	For patients aged between 7 and 11 years All participants played MOON on their home computers after session 10	Any computer with operating system 8/10/11, DirectX 11 support (32 or 64 bits), 1.9-GH processor (Intel core i5 or AMD equivalent), 8 GB RAM, NVIDIA GTX 660 or AMD Radeon HD 7950 with at least 3-GB storage space, and internet connection Controller: mouse and keyboard	Gameplay: the player’s camera depends on the mouse pointer

A PlayStation 4 Dev Kit and Test Kit were used for VR version. The Dev Kit and Test Kit are development consoles equipped with debug firmware that allow the execution of unsigned builds; access to memory and performance analysis tools; real-time error trace logging; as well as software compatibility, optimization, and development testing in a controlled environment. The PlayStation VR for PlayStation 4 is a VR headset featuring a 5.7-inch OLED display with a resolution of 1920×1080 (960×1080 per eye), a field of view of approximately 100°, and a refresh rate of 90/120 Hz. A PlayStation camera was used for motion tracking through an accelerometer, gyroscope, and 9 LEDs. The participants had to be placed at a distance of 1.5 to 2 m with a not very intense light for the correct calibration of the PlayStation camera with the LED lights. The researchers monitored what each participant experienced in VR by viewing it on a computer screen.

The PlayStation 4 VR goggles weigh approximately 600 g, so they could be uncomfortable for some participants. Regarding the added weight of sound equipment, patients were given a choice between basic stereo headphones and over-the-ear headphones. Both had 3D stereo sound and noise cancelation.

A Stealth 15M A11SDK (MSI) laptop was used for participants aged between 7 and 11 years to play MOON in the hospital. The laptop specifications were as follows: Microsoft Windows 11 Home ×64 bits; 11th Gen Intel Core i7-1185G7, 3.00 GHz, 16 GB RAM, 8 GB storage memory; GeForce GTX 1660 Ti.

### Study Outcomes

#### Primary Outcome

The SDQ is a brief 25-item questionnaire that examines difficulties related to emotions, behavior, and social relationships. SDQ includes 5 subscales: emotional symptoms (somatic, worries, unhappy, clingy, and fears), behavioral problems (tempers, not obedient, fights, lies, and steals), hyperactivity (restless, fidgety, distractible, not reflective, and not persistent), peer problems (solitary, not good friend, not popular, bullied, and best with adults), and prosocial behavior (considerate, shares, caring, kind to kids, and helps) [33,34]. The authors propose an alternative three-factor model: (1) prosocial behavior; (2) internalizing difficulties (composed of the subscales of “emotional symptoms” and “peer problems”; cutoff point=7) as an indicator of the presence of excessive worry, somatization with headache, depressive mood, nervousness in unfamiliar situations, and tendency to startle easily; and (3) externalizing difficulties (composed of the subscales “hyperactivity” and “behavior problems”; cutoff point=4), to assess the tendency to lose control, fighting with other children, deceiving, and stealing. The questionnaire has shown adequate psychometric properties in the grouping of these 3 factors and good sensitivity and specificity for detecting children with ADHD [35,36].

The face-to-face web-based change will be measured by comparing the 3 SDQ measures. Subsequently, 3 parent SDQ measures were assessed: initial assessment (D0), midterm assessment (D45), and final assessment (D90) to evaluate hypothesis 5 (switching from face-to-face to web-based methods do not lead to differences in emotional regulation).

#### Secondary Outcomes

The core ADHD symptomatology was measured using 3 subjective scales for parents: the Swanson, Nolan, and Pelham Rating Scale (SNAP-IV); the Conners Abbreviated Symptom Questionnaire (CPRS); and CGI. SNAP-IV [37] is an 18-item questionnaire that assess attention deficit (cutoff point is 1.78) and hyperactive impulsive (cutoff point is 1.44) with a Likert scale ranging from 0 to 4. The CPRS [38] also assesses ADHD symptoms with cutoff points of 16 for boys and 12 for girls. It is a 10-item questionnaire using a Likert scale of 0 to 3 (0=not true at all or never, 1=just a little true or occasionally, 2=pretty much true or often, and 3=very much true or very often). The CGI-40 [39], adapted for parents, allows them to rate their child’s general condition using a Likert scale ranging from 1 to 10.

Executive function refers to a set of top-down processes that allow us to be flexible and to direct behavior toward a goal. Executive function was measured with a parent questionnaire and 3 objective tests for the participants. BRIEF-2 [40] is a 63-item questionnaire with 3 answer options (never, sometimes, and frequently) that provides 4 indices: emotional regulation, cognitive regulation, behavioral regulation, and global index of executive function. For objective testing of patients, we assessed them using the Conners’ Continuous Performance Test Third Edition (CPT-3); Corsi cubes; and the Comprehensive Trail-Making Test, Second Edition (CTMT-2). CPT-3 [41,42] is a computerized, standardized, and validated application test for screening ADHD in clinical practice. CPT-3 provides results on hits, errors of omission, commission error, hit mean reaction time, and variability. Corsi cubes are used to measure visuospatial working memory. CTMT-2 is used to measure cognitive flexibility, with 3 indexes: inhibitory control, task switching, and total index.

Additional school information was collected due to its potential impact on emotional regulation and executive functions. Information about academic performance will be obtained via the patients’ grades.

#### Safety Outcomes

During the development of the video game, risk control measures were applied, especially those related to the possible adverse consequences of VR. We followed recommendations from Sony Interactive Entertainment (Project Morpheus PlayStation Virtual_000010933011) regarding best practices for VR interfaces, including interaction, navigation, user interface, game environments, player comfort, and safety. In addition, risk prevention measures, particularly those related to VR, were implemented (Textbox 1). Before conducting the clinical trial, programming adjustments were made. The game was tested for correct playability using a quality assurance tester. Development was an iterative process using a user-centered model in which game bugs were corrected based on the usability study and the previously conducted clinical trial [28,29].

**Risk prevention and control measures in The Secret Trail of Moon clinical trial.**

**Programming**

- Control latency and frame rate, maintaining a minimum of 90 frames per second. Ensure synchronization between player movements and virtual reality (VR) visuals
- Minimize motion blur
- Maintain a field of view between 90 and 110°
- Avoid artificial or conflicting movements that may cause motion sickness by incorporating static or low-motion scenes where possible
- Reduce loading times and ensure smooth transitions to prevent player discomfort
- Limit intense visual effects that may cause fatigue and provide regular breaks
- Manage proximity and positioning of the user interface
- Control the height of the viewfinder or horizon line

**Optimal playability and adjustment**

- Maintain a player distance of 1.5 m from the PlayStation Camera, with the camera positioned slightly above the eye level to capture head and body movements
- Ensure moderate lighting for better motion detection
- Address visual accommodation: calibrate eye settings and clean lenses regularly
- Assess user comfort, including helmet weight, sound volume, and potential issues such as blurred vision

**Predictable misuse of software and hardware in children and adolescents with attention-deficit/hyperactivity disorder**

- Reduce sudden camera movements with aid of the researcher
- Ensure a clear play space of approximately 2 m around the player, taking special care to manage cables
- Monitor for excessive hyperkinesia or hypokinesia, assessing the potential stress of using new technologies
- Remove VR gear gradually to prevent dizziness
- Evaluate the risk of video game addiction using the Game Addiction Scale for Adolescents and Udvalg für Kliniske Undersogelser (UKU) tests
- Assess fatigue and motivation levels related to gameplay using the UKU test and usability measures
- Monitor drowsiness and arousal levels with the UKU and Sleep Disturbance Scale for Children tests
- Cease use immediately if dizziness occurs. If dizziness arises in ≥2 sessions, consider restricting gameplay to the computer only

Safety outcomes focused on measuring the risk of video game addiction and associated sleep difficulties. Motion sickness may occur with VR, especially in the early adaptation sessions. The group that played MOON with VR was additionally given a measure to control for this possible effect. According to the protocol, the sponsor must notify the Spanish Agency of Medicines and Health Products within 15 calendar days of receiving the Serious Adverse Event report, in the case of any serious adverse events.

The Game Addiction Scale for Adolescents (GASA) is a 7-item questionnaire to assess video game addiction [43,44]. The version of GASA used was the 7-item Spanish version, which measures salience, tolerance, mood modification, relapse, withdrawal, conflict, and problems. A higher score indicates more problematic gaming behavior. Video game addiction is calculated by obtaining a score of ≥3 on at least 4 items or a total score with a cutoff point of 21.

The Sleep Disturbance Scale for Children is a 26-item questionnaire whose subscales measure disorders of initiating and maintaining sleep, sleep breathing disorders, disorders of arousal nightmares, sleep-wake transition disorders, disorders of excessive somnolence, and sleep hyperhydrosis [45]. A higher score indicates more sleep disturbance. Sleep problems are calculated by obtaining a total score with a cutoff point of 39.

Only for MOON group participants aged >12 years and mainly due to the possibility of motion sickness with VR, the questionnaire Udvalg für Kliniske Undersogelser (UKU) was applied after each session. UKU evaluate psychic, neurological, autonomic, and other side effects [46]. The most relevant side effects evaluated with UKU were those associated with ADHD symptoms (concentration difficulties, failing memory, depression, tension, emotional difference, and hyperkinesia), those associated with side effects after using VR (accommodation disturbances, nausea, dizziness, and headache), those associated with the use of video games (physical and psychic dependence), and those associated with sleep (fatigue, sleepiness or sedation, reduced or increased duration of sleep, and increased dream activity).

### Statistical Analyses

#### Sample Size Calculation

To calculate the sample size, we referenced the means of the main measure (SDQ) in similar studies. To account for most possibilities with a significance level of *P*=.05 and a statistical power of 80%, while considering a dropout rate of 15%, the author AR calculated that a total of 152 participants with an ADHD diagnosis were needed. We performed the sample size calculation to contrast a mean decrease of 3 points (symptomatologic improvement) in the experimental group with a SD of 4.

#### Randomization and Masking: Sequence Generation and Allocation

A randomized block sequence of 76 times “number 1 block” (experimental) and 76 times “number 2 block” (control) was generated in 4 blocks of 38 numbers (19 times for number 1 and 19 times for number 2) using the program R (R Foundation for Statistical Computing) by MB-F. The sequence was unknown to the recruiters and was enclosed in the eCRF. The randomization was done through REDCap (eCRF) after the initial evaluation was completed.

#### Statistical Methods

A 2-way ANOVA were performed, with time (pre and post; pre-mid-term-post in the SDQ questionnaire) and group (experimental vs control) as factors and each measure as dependent variable. The difference between the groups was considered significant when *P*<.05. In addition, clinical trends of improvement have been described due to noncompliance with the proposed sample size. These analyses were performed with SPSS (IBM Corp) and R software.

Analyses were performed under the ITT assumption following the protocol; analyses were not performed on PP (only 12 participants completed 20 sessions); an additional analysis has been added regarding the MOON subgroup that completed 80% of the treatment (16 sessions) and, therefore, had a higher involvement in the treatment. As this variable was relevant to the results, an ANCOVA was performed using the number of sessions as a covariate, which had a mean of 18.22 (SD 3.12; range 0-20) sessions. The adjusted means of the groups were calculated based on this mean value.

The ANOVA for hypothesis 4 could not be conducted due to insufficient data. The mean and SD for each academic grade are provided in Multimedia Appendix 2.

## Results

### Recruitment: Patient Inclusion and Randomization

The recruitment period was between May 9, 2023, and October 31, 2023. The enrollment target was not completed (n=152) [30]. The last post evaluation was performed on December 13, 2023. The clinical trial ended on December 15, 2023. A total of 76 patients with ADHD participated in the clinical trial and signed a reported consent form. No patients were excluded. The 76 participants were randomized 1:1 (MOON: n=38, 50% and control: n=38, 50%).

**Figure 1:**
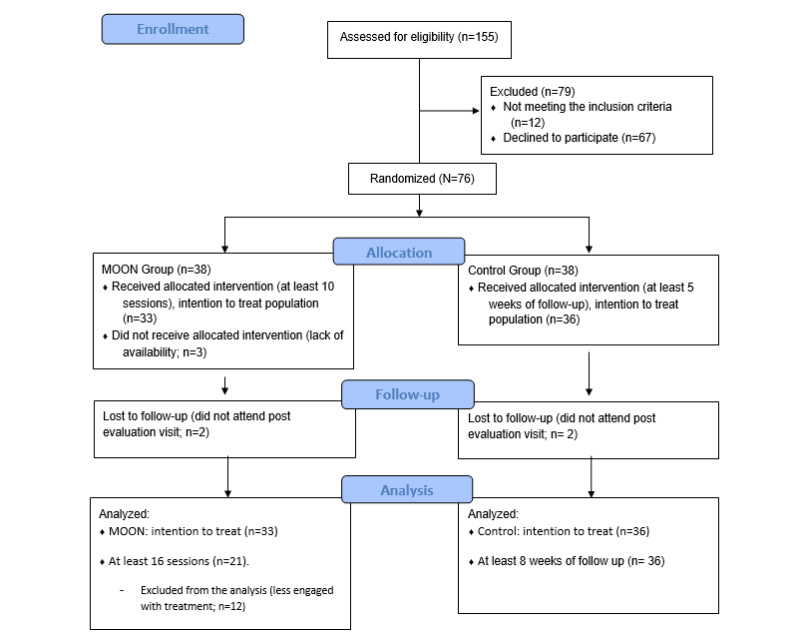
CONSORT (Consolidated Standards of Reporting Trials) flow diagram for the number of participants in the clinical trial. ITT: intention-to-treat; MOON: The Secret Trail of Moon.

The total dropout rate was 9% (7/76) of the participants (n=5, 71% MOON and n=2, 29% control) and did not exceed expectations (12/76, 15%). Lack of availability was the reason mentioned by the dropout cases for not participating. In total, 3% (2/38) of the patients requested to switch to the control group after preassessment and randomization to MOON due to insufficient time to commit to the study, which was not allowed. Another MOON participant had to drop out of treatment due to lack of availability after 2 sessions. In total, 5% (4/76) of the participants (n=2, 50% MOON and n=2, 50% control) were not assessed at postevaluation and were lost to follow-up (Figure 1). The ITT population was analyzed for the primary and secondary outcomes. The ITT population included 87% (33/38) of the patients assigned to the MOON group and 95% (36/38) of the patients assigned to the control group.

### Baseline Characteristics

Table 2 shows the sociodemographic variables for all participants who completed a preevaluation (N=76). The mean age of the groups were 12.79 (SD 2.86) years for MOON and 12.58 (SD 2.67) years for control. In total, 80% (61/76) of the sample was composed of male participants.

Regarding multimodal treatment, 30% (23/76) of the participants were receiving >1 medication per day. The remaining 70% (53/76) were taking only their ADHD pharmacological treatment. As much as possible, ADHD patients were instructed not to change their medication during the study, except for clinical reasons (eg, side effects or inadequate medication adjustment at the time of examination). For ethical reasons, the needs of the patients were prioritized, and there were 11 changes: 8 dose increases of the usual medication and 3 dose decreases of the usual medication. Of the abovementioned changes, 3 occurred in the MOON group (1 decrease and 2 dose increases) and 8 occurred in the control group (2 decreases and 6 dose increases). Currently, 41% (30/73) of the participants are receiving psychological treatment, and 30% (22/73) had received it before the study. In addition, all participants received weekly psychoeducation and counseling on ADHD.

School, social, and recreational data have been included in Table 3. Regarding difficulties with peers, 19% (14/76) of the participants experienced bullying and 31% (23/76) had difficulty making friends, as reported by parents. Additional clinical information was collected. Regarding medical history, 45% (34/76) of the mothers had difficulties during pregnancy. Regarding the developmental alterations of the participants, 26% (20/76) had delayed speech development, 15% (11/76) had slow gait development, and 24% (18/76) had delayed sphincter control. Of the participants, 35% (26/76) needed previous speech therapy support, 9% (7/76) needed psychomotor therapy, and 11% (8/76) needed early stimulation.

The results are listed in the primary and secondary outcome sections, ordered according to the hypotheses. Analysis was performed with the ITT population (69/76, 91%) and patients who completed 80% of treatment (57/76, 75%).

**Table 2:** Baseline demographic variables.

Baseline variables			Total (N=76)		MOON^a^ (n=38)		Control^b^ (n=38)		Chi-square (*df*)			*t* test (*df*)			*P* value	
Sex (female), n (%)			15 (20)		7 (18)		8 (21)		0.0 (1)			N/A^c^			.77	
Age (y), mean (SD)			12.68 (2.75)		12.79 (2.86)		12.58 (2.67)		N/A			0.3 (74)			.74	
**Nationality of birth, n (%)**										0.3 (2)			N/A			.84
	European (Spain)	71 (93)		35 (92)		36 (95)										
	Hispanic America	2 (3)		1 (3)		1 (3)										
	Asian	3 (4)		2 (5)		1 (3)										
Adopted, n (%)			4 (5)		2 (5)		2 (5)		—^d^			—			—	
**Family background, n (%)**																
	Lives with both parents	63 (83)		34 (90)		29 (76)		2.3 (1)			N/A			.12		
	Lives with mother	12 (16)		4 (11)		8 (21)		1.5 (1)			N/A			.20		
**Family socioeconomic status (**€ **per month; €1=US $1.4), n (%)**										0.4 (3)			N/A			.93
	500-1500	5 (7)		2 (5)		3 (8)										
	1500-2000	7 (9)		4 (11)		3 (8)										
	2000-2500	13 (17)		6 (16)		7 (18)										
	>2500	51 (67)		26 (68)		25 (66)										

^a^MOON: The Secret Trail of Moon (the experimental group with the serious video game).

^b^Control: control group without the serious video game.

^c^N/A: not applicable.

^c^Not available.

**Table 3:** Clinical characteristics of each group.

Clinical variables			Total (N=76), n (%)	MOON^a^ (n=38), n (%)		Control (n=38), n (%)	Chi-square (*df*)		*P* value	
**Medical history**										
	**ADHD^b^ subtypes**									
		Combined	48 (66)		23 (62)	25 (69)	0.4 (1)		.51	
		Primarily inattentive	25 (34)		14 (37)	11 (31)	—^c^		—	
	**Medication**									
		Elvanse	53 (55)		29 (62)	24 (49)	8.5 (10)		.58	
		Equasym	8 (9)		3 (7)	5 (11)	—		—	
		Intuniv	7 (7)		3 (6)	4 (8)	—		—	
		Medikinet	5 (5)		2 (4)	3 (6)	—		—	
		Risperdal	6 (6)		2 (4)	4 (8)	—		—	
		Concerta	3 (3)		1 (2)	2 (4)	—		—	
		Other: atenza, atomoxetina, rubifen retard, and rubicrono	4 (4)		2 (4)	2 (4)	—		—	
		Invega and zyprexa	2 (2)		1 (2)	1 (2)	—		—	
		Fluoxetina and sertralina	8 (9)		4 (9)	4 (8)	—		—	
**Psychological therapy**								1.2 (2)		.52
	Current		30 (41)	13 (34)		9 (25)				
	Previous		22 (29)	13 (34)		17 (47)				
**School background**										
	Repeating a school year		21 (8)	11 (29)		10 (27)	0.3 (1)		.85	
	Special education		8 (11)	3 (8)		5 (14)	0.6 (1)		.43	
	Curricular adaptation		35 (47)	21 (55)		14 (37)	2.2 (1)		.13	
	Private teacher		24 (32)	10 (26)		14 (37)	1.1 (1)		.28	
	High abilities program		6 (8)	5 (13)		1 (3)	2.7 (1)		.09	
**Peer problems**										
	Bullying		14 (19)	4 (11)		10 (26)	3.3 (1)		.06	
	Tendency to isolate		14 (19)	7 (18)		7 (19)	0.0 (1)		.95	
	Difficulty in making friends		23 (31)	10 (26)		13 (35)	0.6 (1)		.40	
	Behavioral problems		13 (17)	5 (13)		8 (22)	0.9 (1)		.33	
**Recreation**										
	Sports		60 (79)	30 (83)		30 (88)	—		—	
	Play a musical instrument		11 (14)	5 (13)		4 (11)	—		—	
	**Regular video game use (h)**		53 (74)	28 (76)		25 (71)	0.1 (2)		.68	
		0.25-1	21 (44)		10 (39)	11 (50)	0.9 (1)		.62	
		1.5-2	14 (29)		9 (35)	5 (23)	—		—	
		2.5-5	13 (27)		7 (18)	6 (27)	—		—	
	**Regular mobile use (h)**		50 (67)	—		—	—		—	
		0.25-1	10 (24)		6 (27)	4 (20)	0.8 (2)		.66	
		1.5-2	16 (38)		7 (32)	9 (45)	—		—	
		2.5-6	16 (38)		9 (41)	7 (35)	—		—	

^a^MOON: The Secret Trail of Moon.

^b^ADHD: attention-deficit/hyperactivity disorder.

^c^Not available.

### Primary Outcome: Emotional Regulation

The main hypothesis of a 3- or 4-point drop in the primary end point of global SDQ was not achieved. We found no statistically significant differences in the primary variable (SDQ) in our pretest and posttest comparison (*F*_1,64_=0.51; *P*=.47; η^2^=0.00; 1–β=0.11). The preassessment overall SDQ scores for the experimental group (mean 27.48, SD 5.64; 95% CI 25.45-29,51) and the control group (mean 26.61, SD 6.03; 95% CI 24.57-28.63) were higher than the post scores of the experimental group (mean 26.09, SD 5.74; 95% CI 24.06-28.12) and the post scores of the control group (mean 25.94, SD 5.93; 95% CI 23.90-27.97). Significant differences were found in the prosocial scale in favor of the control group (*F*_1, 66_=3.73; *P*=.05; η^2^=0.05; 1–β=0.47). These results are shown in Table 4 and Multimedia Appendix 3.

**Table 4:** Primary outcome: pre-post comparison of the Strengths and Difficulties Questionnaire (SDQ) scores.

				Emotional symptoms		Behavior problems		Hyperactivity		Peer problems		Prosocial scale		Internalizing		Externalizing		SDQ global	
**Intention-to-treat (at least 10 sessions; n=69)**																			
	**MOON^a^ (n=33), mean (SD)**																		
		Pre	9.18 (2.54)		6.39 (1.56)		8.03 (2.00)		3.88 (2.53)		12.18 (2.37)		13.06 (4.39)		14.42 (2.68)		27.48 (5.64)		
		Post	8.85 (2.19)		6.27 (1.66)		7.27 (2.28)		3.70 (2.55)		11.78 (2.56)		12.55 (4.10)		13.55 (3.15)		26.09 (5.74)		
	**Control (n=36), mean (SD)**																		
		Pre	9.00 (2.44)		6.19 (1.93)		7.83 (2.39)		3.91 (2.35)		12.78 (1.88)		12.86 (4.04)		14.09 (3.52)		26.79 (6.03)		
		Post	8.89 (2.23)		6.08 (1.85)		7.33 (2.49)		4.00 (2.60)		13.08 (1.76)		12.74 (3.99)		13.42 (3.82)		26.09 (5.91)		
	*F* test (*df*)		0.50 (1)		0 (1)		0.40 (1)		0.70 (1)		3.73 (1)		0.96 (1)		0.21 (1)		0.51 (1)		
	*P* value		.48		.97		.52		.40		.05		.33		.64		.47		
	η^2^		0.00		0.00		0.00		0.01		0.05		0.01		0.00		0.00		
**16 sessions (n=57)**																			
	**MOON (n=21), mean (SD)**																		
		Pre	9.67 (2.45)		6.57 (1.56)		7.90 (1.84)		4.24 (2.54)		11.33 (2.24)		13.90 (4.19)		14.48 (2.54)		28.38 (5.54)		
		Post	9.00 (2.12)		6.33 (1.56)		7.38 (2.24)		3.76 (2.36)		11.14 (2.15)		12.76 (3.83)		13.71 (3.28)		26.48 (5.76)		
	**Control (n=36), mean (SD)**																		
		Pre	9.00 (2.44)		6.19 (1.93)		7.83 (2.39)		3.91 (2.35)		12.78 (1.88)		12.86 (4.04)		14.09 (3.52)		26.79 (6.03)		
		Post	8.89 (2.23)		6.08 (1.85)		7.33 (2.49)		4.00 (2.60)		13.08 (1.76)		12.74 (3.99)		13.42 (3.82)		26.09 (5.91)		
	*F* test (*df*)		1.95 (1)		0.10 (1)		0.02 (1)		2.28 (1)		1.85 (1)		3.35 (1)		0.05 (1)		1.08 (1)		
	*P* value		.16		.75		.87		.13		.17		.07		.81		.30		
	η^2^		0.03		0.00		0.00		0.04		0.03		0.05		0.00		0.02		

^a^MOON: *The Secret Trail of Moon*.

In the most engaged subgroup (16 sessions), clinical trends of improvement on the SDQ of 2 points were observed but did not approach significance. This improvement trend is particularly notable in internalizing difficulties (*F*_1, 53_=3.35; *P*=.07; η^2^=0.05; 1–β=0.43), peer problems (*F*_1, 54_=2.28; *P*=.13; η^2^=0.04; 1–β=0.31) and emotional symptoms (*F*_1, 54_=1.95; *P*=.16; η^2^=0.03; 1–β=0.27; Table 4; Multimedia Appendix 3). At the SDQ follow-up, parents in the MOON group reported that their children felt “a little better,” while parents in the control group reported their children felt “about the same.”

All participants completed the 10 face-to-face sessions. However, the number of sessions decreased when there was no direct face-to-face supervision by the investigators in the MOON web-based intervention. The results of the main variable (SDQ) of the 3 measures (pre-mid-term-post comparison) are shown in Table 5 and Multimedia Appendix 4. The change of modality (face-to-face and on the web) could have influenced adherence to treatment. The MOON group had an average of 16 sessions, with 3 participants having the minimum number of sessions (10) and 12 patients having the maximum number (20; Multimedia Appendix 5). Most participants in the MOON group (26/33, 79%) played the video game in VR, as they were aged >12 years, following PlayStation’s recommendations. A comparative statistical analysis was conducted within the MOON group, comparing participants who played the face-to-face VR version (26/33, 79%) with those who used the computer version for the entire treatment (7/33, 21%). We found that the VR subgroup improved most on emotional symptoms (*F*_1, 31_=7.79; *P*=.004; η^2^=0.20; 1–β=0.77), internalizing (*F*_1, 31_=4.18; *P*=.04; η^2^=0.11; 1–β=0.50) and, to a lesser extent, global SDQ (*F*_1, 31_=2.99; *P*=.09; η^2^=0.08; 1–β=0.38).

**Figure 2:**
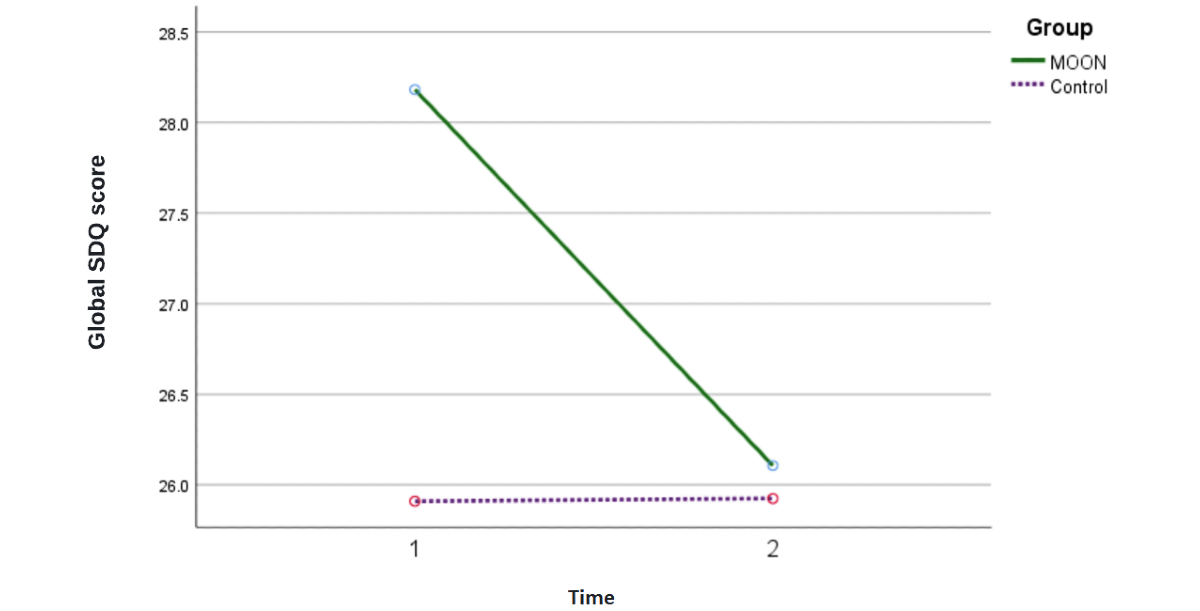
Results of analysis of covariance of the main hypothesis (decrease in The Secret Trail of Moon [MOON] group of 3 or 4 points in global Strengths and Difficulties Questionnaire [SDQ] compared to the control group). The number of sessions was used as a covariate.

The ANCOVA for the main hypothesis—which posited that the MOON group would exhibit improved emotional regulation, reflected by a 3- to 4-point decrease in global SDQ scores compared to the control group—was conducted with the number of sessions included as a covariate. The results approached significance (*F*_1, 63_=3.25; *P*=.07; η^2^=0.04; 1–β=0.42; Figure 2; Table 6). Significant improvements were found in the MOON group in the variables of emotional symptoms (*F*_1, 65_=4.47; *P*=.03; η^2^=0.06; 1–β=0.54) and internalizing problems (*F*_1, 64_=6.57; *P*=.01; η^2^=0.09; 1–β=0.71).

**Table 5:** Primary outcome: pre-mid-term-post comparison of the Strengths and Difficulties Questionnaire (SDQ) scores.

					Emotional symptoms			Behavior problems			Hyperactivity			Peer problems			Prosocial scale			Internalizing			Externalizing			SDQ global	
**Intention-to-treat (at least 10 sessions; n=69)**																											
	**MOON^a^ (n=33),mean (SD)**																										
		Pre	9.18 (2.54)			6.39 (1.56)			8.03 (2)			3.88 (2.53)			12.18 (2.37)			13.06 (4.39)			14.42 (2.68)			27.48 (5.64)			
		Midterm	8.91 (2.28)			6.09 (1.52)			7.58 (2.18)			3.88 (2.80)			12.06 (2.46)			12.79 (4.38)			13.67 (2.88)			26.45 (5.71)			
		Post	8.85 (2.19)			6.27 (1.66)			7.27 (2.28)			3.70 (2.55)			11.78 (2.56)			12.55 (4.10)			13.55 (3.15)			26.09 (5.74)			
	**Control (n=36),** **m** **ean (SD)**																										
		Pre	9 (2.44)			6.19 (1.93)			7.83 (2.39)			3.91 (2.35)			12.78 (1.88)			12.86 (4.04)			14.09 (3.52)			26.79 (6.03)			
		Midterm	8.69 (2.30)			6.25 (2.08)			6.97 (2.55)			3.97 (2.43)			12.72 (1.95)			12.67 (3.89)			13.22 (3.72)			25.89 (5.80)			
		Post	8.89 (2.23)			6.08 (1.85)			7.33 (2.49)			4 (2.60)			13.08 (1.76)			12.74 (3.99)			13.42 (3.82)			26.09 (5.91)			
	*F* test (*df*)			0.44 (2)			0.71 (2)			0.94 (2)			0.72 (2)			2.33 (2)			0.86 (2)			0.13 (2)			0.39 (2)		
	*P* value			.64			.49			.39			.48			.10			.42			.87			.67		
	η^2^			0			0.01			0.01			0.01			0.03			0.01			0			0		
**16 sessions (n=57)**																											
	**MOON (n=21), mean (SD)**																										
		Pre	9.67 (2.45)			6.57 (1.56)			7.90 (1.84)			4.24 (2.54)			11.33 (2.24)			13.90 (4.19)			14.48 (2.54)			28.38 (5.54)			
		Midterm	9.29 (2.10)			6.10 (1.37)			7.57 (1.96)			4.05 (2.90)			11.76 (2.11)			13.33 (4.16)			13.62 (2.72)			26.95 (5.81)			
		Post	9 (2.12)			6.33 (1.56)			7.38 (2.24)			3.76 (2.36)			11.14 (2.15)			12.76 (3.83)			13.71 (3.28)			26.48 (5.76)			
	**Control (n=36), mean (SD)**																										
		Pre	9 (2.44)			6.19 (1.93)			7.83 (2.39)			3.91 (2.35)			12.78 (1.88)			12.86 (4.04)			14.09 (3.52)			26.79 (6.03)			
		Midterm	8.69 (2.30)			6.25 (2.08)			6.97 (2.55)			3.97 (2.43)			12.73 (1.95)			12.67 (3.89)			13.22 (3.72)			25.89 (5.80)			
		Post	8.89 (2.23)			6.08 (1.85)			7.33 (2.49)			4 (2.60)			13.08 (1.76)			12.74 (3.99)			13.42 (3.82)			26.09 (5.91)			
	*F* test (*df*)			1.30 (2)			1.01 (2)			0.45 (2)			1.51 (2)			3.15 (2)			2.41 (2)			0.03 (2)			0.65 (2)		
	*P* value			.27			.36			.63			.22			*.04*			.09			.96			.51		
	η^2^			0.02			0.01			0			0.02			0.05			0.04			0			0.01		

^a^MOON: *The Secret Trail of Moon*.

**Table 6:** Analysis of covariance of the Strengths and Difficulties Questionnaire (SDQ) using the number of sessions as a covariate (N=69).

SDQ	Intention-to treat (at least 10 sessions)							
	MOON^a^ (n=33), mean (SD)		Control (n=36), mean (SD)		*F* test (*df*)	*P* value	η^2^	1–β
	Pre	Post	Pre	Post				
Emotional symptoms	9.18 (2.54)	8.85 (2.19)	8.91 (2.43)	8.89 (2.23)	4.47 (1)	.03	0.06	0.54
Behavior problems	6.39 (1.56)	6.27 (1.66)	6.19 (1.93)	6.08 (1.85)	0.32 (1)	.56	0	0.08
Hyperactivity	8.03 (2.00)	7.27 (2.28)	7.83 (2.39)	7.40 (2.49)	0.43 (1)	.51	0	0.10
Peer problems	3.88 (2.53)	3.70 (2.55)	3.91 (2.35)	4.00 (2.60)	2.74 (1)	.10	0.04	0.37
Prosocial scale	12.09 (2.36)	11.78 (2.56)	12.78 (1.88)	13.08 (1.76)	1.19 (1)	.27	0.01	0.19
Internalizing	13.06 (4.39)	12.55 (4.10)	12.68 (3.96)	12.74 (3.99)	6.57 (1)	*.01*	0.09	0.71
Externalizing	14.42 (2.68)	13.55 (3.15)	14.09 (3.52)	13.51 (3.83)	0.57 (1)	.44	0	0.11
SDQ global	28.18 (1.10)	26.10 (1.12)	25.90 (1.10)	25.94 (1.12)	3.25 (1)	.07	0.04	0.42

^a^MOON: *The Secret Trail of Moon*.

### Secondary Outcomes

#### Core ADHD Symptoms

No significant differences (*P*>.05) were found in the main ADHD symptoms in SNAP-IV and CPRS.

Clinical trends of improvement were seen in the MOON group versus control in the subscales of hyperactivity (*F*_1, 66_=3.06; *P*=.08; η^2^=0.04) and inattention (*F*_1, 66_=2.41; *P*=.12; η^2^=0.03) and in the total scale of SNAP-IV (F_1, 67_=2.63; *P*=.11; η^2^=0.03) and CPRS (*F*_1, 65_=2.06; *P*=.15; η^2^=0.03). In the participants who more engaged in the MOON treatment (at least 16 sessions), clinical trends of improvement were also observed in hyperactivity (*F*_1, 54_=2.51; *P*=.11; η^2^=0.04) and CPRS total scale (*F*_1, 53_=2.05; *P*=.15; η^2^=0.03; Table 7; Multimedia Appendix 6).

**Table 7:** Secondary outcomes related to hypotheses 2 and 3 (patients with attention-deficit/hyperactivity disorder [ADHD] using The Secret Trail of Moon [MOON] improve in core ADHD symptoms [hypothesis 2] and cognitive functioning compared with the control group; N=69).

				Intention-to-treat (at least 10 sessions; n=69)																				16 sessions (n=57)																
				MOON (n=33), mean (SD)					Control (n=36), mean (SD)					*F* test (*df*)			*P* value			ηp^2^			MOON (n=21), mean (SD)						Control (n=36), mean (SD)					*F* test (*df*)			*P* value			ηp^2^
				Pre		Post		Pre			Post											Pre				Post		Pre			Post									
**Core ADHD**																																								
	**SNAP-IV^a^**																																							
		Inattention	28.73 (5.51)		25.91 (6.44)		26.67 (6.59)			25.78 (6.06)		2.41 (1)			.12			0.03			28.05 (6.16)				26.33 (6.32)		26.67 (6.59)			25.78 (6.06)		0.37 (1)			.54			0.00		
		Hyperactivity	23.12 (5.93)		20.18 (5.37)		23.42 (7.81)			21.91 (7.50)		3.06 (1)			.08			0.04			24.43 (5.10)				21.33 (4.99)		23.42 (7.81)			21.91 (7.50)		2.51 (1)			.11			0.04		
		Total	51.85 (8.96)		45.30 (10.97)		50.08 (12.69)			47.08 (11.88)		2.63 (1)			.11			0.03			52.48 (8.86)				47.67 (9.11)		50.08 (12.69)			47.08 (11.88)		0.53 (1)			.46			0.01		
	**CPRS^b^**																																							
		ADHD symptoms	26.67 (5.17)		23.97 (6.02)		25.23 (6.17)			23.86 (6.60)		2.06 (1)			.15			0.03			27.95 (4.45)				25.00 (5.74)		25.23 (6.17)			23.86 (6.60)		2.05 (1)			.15			0.03		
	**CGI^c^**																																							
		General condition rate	6.9 (11.28)		7.1 (12.65)		6.6 (15.59)			6.7 (12.55)		0.13 (1)			.71			0.00			7.2 (9.82)				7.0 (10.94)		6.6 (15.59)			6.7 (12.55)		0.25 (1)			.61			0.00		
**Cognitive functioning**																																								
	**BRIEF-2^d^**																																							
		Inhibition	66.00 (12.64)		63.09 (12.60)		63.92 (12.19)			63.67 (12.86)		1.79 (1)			.18			0.02			68.48 (12.17)				63.81 (12.47)		63.92 (12.19)			63.67 (12.86)		3.89 (1)			.05			0.06		
		Self-supervision	66.58 (12.20)		63.76 (12.52)		63.19 (10.07)			61.14 (14.18)		0.17 (1)			.68			0.00			68.76 (11.36)				65.43 (12.08)		63.19 (10.07)			61.14 (14.18)		0.17 (1)			.68			0.00		
		Flexibility	69.00 (16.96)		67.82 (16.18)		66.81 (15.00)			65.86 (17.41)		0 (1)			.93			0.00			72.67 (16.53)				70.90 (15.46)		66.81 (15.00)			65.86 (17.41)		0.05 (1)			.80			0.00		
		Emotional control	63.45 (12.07)		59.76 (11.65)		62.25 (10.91)			61.03 (11.08)		1.25 (1)			.26			0.01			66.24 (10.09)				61.86 (10.72)		62.25 (10.91)			61.03 (11.08)		1.51 (1)			.22			0.02		
		Initiative	65.85 (11.71)		64.18 (12.38)		59.39 (11.76)			59.86 (13.68)		0.90 (1)			.34			0.01			65.62 (12.91)				65.62 (13.17)		59.39 (11.76)			59.86 (13.68)		0.03 (1)			.85			0.00		
		Working memory	71.27 (10.15)		66.36 (12.40)		67.22 (11.52)			65.94 (12.90)		3.49 (1)			.06			0.05			71.48 (11.69)				64.33 (16.17)		67.22 (11.52)			65.94 (12.90)		4.08 (1)			.04			0.06		
		Planning and organization	67.88 (9.83)		66.52 (11.29)		62.61 (11.21)			62.39 (11.70)		0.49 (1)			.48			0.00			68.62 (10.70)				67.14 (11.72)		62.61 (11.21)			62.39 (11.70)		0.56 (1)			.45			0.01		
		Task supervision	65.36 (11.92)		62.82 (13.00)		62.47 (10.12)			61.03 (12.50)		0.40 (1)			.52			0.00			64.57 (13.73)				61.95 (13.82)		62.47 (10.12)			61.03 (12.50)		0.44 (1)			.65			0.00		
		Material organization	73.76 (13.01)		71.03 (12.71)		65.44 (13.96)			64.36 (16.19)		0.61 (1)			.43			0.00			75.95 (13.23)				70.00 (12.16)		65.44 (13.96)			64.36 (16.19)		4.75 (1)			.03			0.08		
		Behavioral regulation Index	67.73 (12.38)		64.70 (12.95)		64.92 (12.25)			63.94 (12.41)		1.06 (1)			.30			0.01			70.29 (11.77)				65.95 (12.72)		64.92 (12.25)			63.94 (12.41)		2.29 (1)			.13			0.04		
		Emotional regulation Index	68.55 (15.28)		65.30 (13.97)		66.39 (12.27)			64.97 (13.78)		0.64 (1)			.42			0.01			72.14 (14.01)				68.10 (12.19)		66.39 (12.27)			64.97 (13.78)		0.96 (1)			.33			0.01		
		Cognitive regulation index	72.64 (11.31)		69.36 (12.98)		66.28 (11.93)			65.56 (13.90)		2.52 (1)			.11			0.03			73.10 (13.09)				69.33 (13.92)		66.28 (11.93)			65.56 (13.90)		3.42 (1)			.07			0.05		
		Global executive function	75.88 (16.29)		70.39 (13.13)		71.44 (17.28)			70.44 (19.40)		3.56 (1)			.06			0.03			76.05 (11.83)				71.38 (12.87)		71.44 (17.28)			70.44 (19.40)		3.58 (1)			.06			0.06		
	**CPT-3^e^**																																							
		Detectability	45.97 (11.71)		46.88 (13.11)		47.67 (8.70)			45.25 (9.35)		3.29 (1)			.07			0.04			46.81 (11.38)				49.57 (13.60)		47.67 (8.70)			45.25 (9.35)		5.92 (1)			.01			0.09		
		Omissions	50.67 (13.62)		52.27 (15.08)		49.00 (8.07)			48.97 (11.69)		0.43 (1)			.51			0.00			52.95 (15.99)				56.10 (17.68)		49.00 (8.07)			48.97 (11.69)		1.08 (1)			.30			0.01		
		Commissions	44.73 (9.78)		45.33 (10.29)		46.25 (8.65)			43.97 (7.77)		2.92 (1)			.09			0.04			43.81 (8.75)				46.00 (10.20)		46.25 (8.65)			43.97 (7.77)		5 (1)			.02			0.08		
		Perseverations	51.55 (11.57)		54.15 (15.76)		50.64 (10.97)			50.11 (8.71)		1.07 (1)			.30			0.01			52.67 (13.39)				58.10 (18.55)		50.64 (10.97)			50.11 (8.71)		2.66 (1)			.10			0.04		
		HRT^f^	57.76 (12.82)		56.91 (11.14)		56.22 (8.58)			58.19 (10.95)		2.61 (1)			.11			0.03			59.81 (13.67)				58.76 (11.47)		56.22 (8.58)			58.19 (10.95)		1.98 (1)			.16			0.03		
		HRTsd^g^	51.21 (14.38)		53.52 (15.64)		51.19 (9.21)			51.56 (11.98)		0.63 (1)			.43			0.00			53.90 (16.18)				58.81 (16.28)		51.19 (9.21)			51.56 (11.98)		2.49 (1)			.12			0.04		
		Variability	50.69 (12.80)		49.52 (9.87)		51.53 (10.19)			50.69 (8.77)		0.40 (1)			.52			0.00			50.90 (13.73)				52.53 (11.00)		51.53 (10.19)			50.69 (8.77)		0 (1)			.93			0.00		
		Block change	48.28 (8.69)		50.73 (9.84)		50.42 (10.09)			52.08 (12.43)		0.03 (1)			.85			0.00			48.45 (10.25)				51.52 (12.01)		50.42 (10.09)			52.08 (12.43)		0.08 (1)			.76			0.00		
		Inter stimulus change	52.67 (12.77)		57.30 (13.44)		51.97 (10.31)			54.14 (12.01)		1.25 (1)			.26			0.01			55.67 (14.06)				61.81 (13.83)		51.97 (10.31)			54.14 (12.01)		2.20 (1)			.14			0.03		
	**Corsi**																																							
		N span (items recalled)	5.66 (1.15)		5.79 (1.21)		5.17 (0.98)			5.03 (1.20)		0.38 (1)			.53			0.00			5.55 (1.35)				5.67 (1.35)		5.17 (0.98)			5.03 (1.20)		0.27 (1)			.60			.00		
	**CTMT-2^h^**																																							
		Inhibitory control	44.24 (14.41)		52.33 (15.42)		46.06 (13.77)			53.72 (13.62)		0.15 (1)			.69			0.00			41.43 (15.66)				50.95 (17.40)		46.06 (13.77)			53.72 (13.62)		0.74 (1)			.39			.01		
		Set shifting	43.55 (16.72)		52.24 (15.33)		45.60 (12.79)			49.97 (12.09)		4.06 (1)			.04			0.05			41.24 (17.19)				50.00 (16.15)		45.60 (12.79)			49.97 (12.09)		3.05 (1)			.08			.05		
		Total	43.82 (14.86)		52.76 (15.59)		45.74 (12.37)			52.39 (11.82)		1.95 (1)			.16			0.02			41.19 (15.58)				51.00 (17.33)		45.74 (12.37)			52.39 (11.82)		2.45 (1)			.12			.04		

^a^SNAP-IV: Swanson, Nolan, and Pelham Rating Scale.

^b^CPRS: Conners Abbreviated Symptom Questionnaire.

^c^CGI: Clinical Global Impression.

^d^BRIEF: Behavior Rating Inventory Executive Function, version 2.

^e^CPT-3: Conners’ Continuous Performance Test Third Edition.

^f^HRT: Hit Reaction Time.

^g^HRTsd: Hit Reaction Time SD.

^h^CTMT-2: Comprehensive Trail-Making Test, Second Edition.

#### Cognitive Functioning

In the BRIEF-2 questionnaire evaluated by parents, statistically significant differences were observed in the MOON group that was more involved (at least 16 sessions) in material organization (*F*_1, 55_=4.75; *P*=.03; η^2^=0.08), working memory (*F*_1, 55_=4.08; *P*=.05; η^2^=0.06), and inhibition (*F*_1, 55_=3.89; *P*=.05; η^2^=0.06). Some statistically significant differences were also found in the cognitive tests performed by the participants. In the CPT-3 test, improvements in detectability were found (*F*_1, 55_=5.92; *P*=.01; η^2^=0.09) in MOON group; however, significant differences in commissions (*F*_1, 55_=5.00; *P*=.02; η^2^=0.08) were found in favor of the control group; in the CTMT-2 test, an improvement in the MOON group was found in set shifting (*F*_1, 66_=4.06; *P*=.04; η^2^=0.05; Table 7; Multimedia Appendices 6 and 7).

Clinical trends of improvement with MOON versus control (ITT=69) were observed in working memory (*F*_1, 67_=3.49; *P*=.06; η^2^=0.05), inhibition (*F*_1, 67_=1.79; *P*=.18; η^2^=0.02), cognitive regulation index (*F*_1, 67_=2.52; *P*=.11; η^2^=0.03), and global executive function (*F*_1, 6_=3.56; *P*=.06; η^2^=0.03).

#### Side Effects

There were no interruptions due to adverse events. None of the participants had side effects related to the VR treatment lasting >1 hour after use. In total, 2 patients reported discomfort from the weight of the VR headset, so the researchers offered the possibility of using the computer game.

We found no significant differences in video game addiction between the groups assessed with the GASA questionnaire.

**Figure 3:**
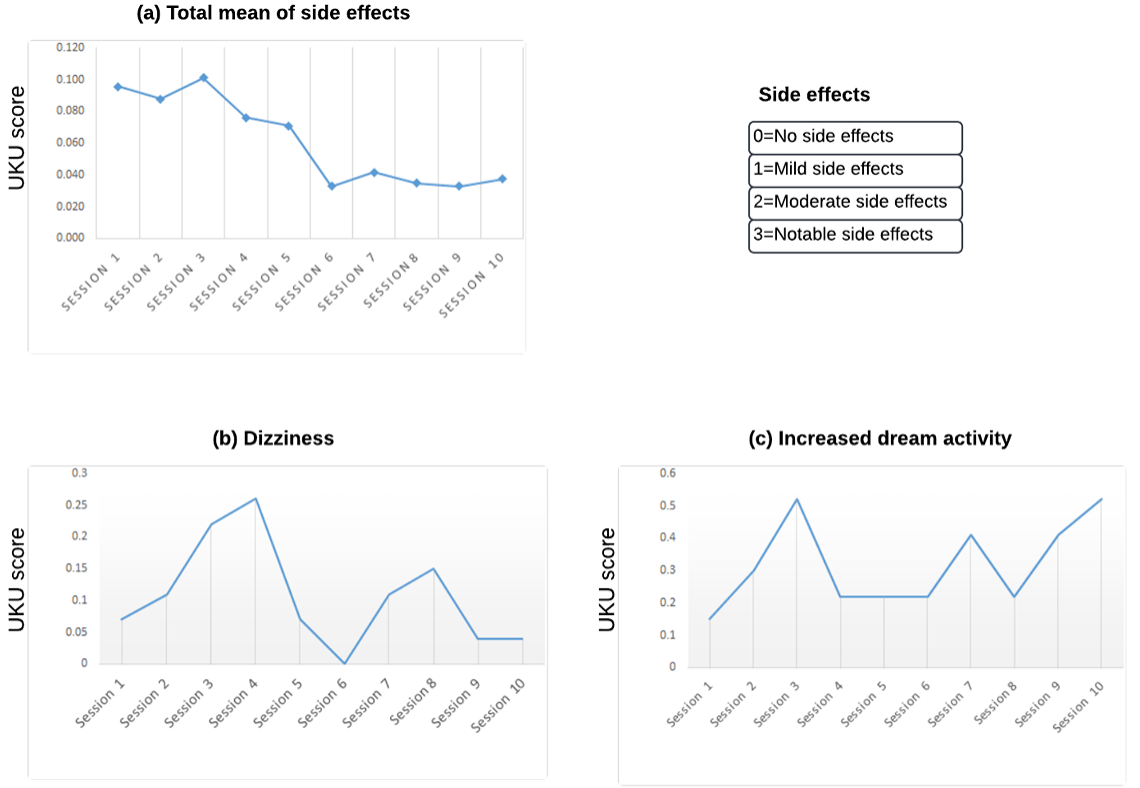
(A) Total mean of the side effects per session, specifically for the most relevant side effects: (B) dizziness (mean 0.10) and (C) increased dream activity (mean 0.31). UKU: Udvalg für Kliniske Undersogelser.

No mild, moderate, or notable side effects were found (Multimedia Appendix 8). Patients who described a causal relationship with the video game through the UKU questionnaire (possible or probable) reported dizziness and increased dream activity (Figure 3; Multimedia Appendices 9 and 10). The mean score for dizziness across the 10 sessions was 0.10 (SD 0.08); the mean score for increased dream activity was 0.31 (SD 0.13; 0=no side effects and 1=mild side effects that do not interfere with patient performance) and was decreasing across sessions; however, some participants (15/26, 58%) reported increased dream activity across sessions.

In relation to sleep problems measured with the Sleep Disturbance Scale for Children questionnaire, no significant differences were found in the number of hours slept (mean in both groups=8-9 h) or in the time taken to fall asleep (mean 15-30 min). Nonetheless, an improvement in favor of the MOON group was found in the disorders of excessive somnolence scale (*F*_1, 65_=4.55; *P*=.03; η^2^=0.06; Table 8).

**Table 8:** Secondary outcomes related to hypothesis 6: there are no clinically meaningful side effects associated with the video game (N=69).

Safety outcomes		Intention-to-treat (at least 10 sessions)							
		MOON^a^ (n=33), mean (SD)		Control (n=36), mean (SD)			*F* test (*df*)		*P* value
		Pre	Post	Pre	Post				
Game addiction (GASA^b^)		17.42 (5.75)	15.70 (5.48)	17.06 (5.25)	15.89 (5.24)	0.32 (1)		.57	
**Sleep disorders (SDSC^c^)**									
	Sleeping hours	2.37 (0.94)	2.39 (0.65)	2.33 (0.92)	2.50 (0.56)	0.57 (1)		.44	
	Time to sleep	2.52 (1.30)	2.15 (0.79)	2.38 (1.24)	2.05 (0.71)	0.00 (1)		.99	
	DIMS^d^	15.53 (4.70)	15.72 (4.60)	15.24 (5.97)	14.74 (5.62)	0.04 (1)		.83	
	SBD^e^	4.13 (1.99)	3.87 (1.47)	4.22 (1.80)	4.36 (1.93)	1.70 (1)		.19	
	DA^f^	4.38 (1.86)	4.15 (1.58)	3.97 (1.40)	3.86 (1.24)	0.13 (1)		.71	
	SWTD^g^	11.88 (4.15)	11.56 (3.78)	11.37 (4.56)	11.64 (5.05)	0.18 (1)		.67	
	DOES^h^	12.09 (4.05)	11.09 (3.97)	9.51 (3.28)	10.00 (4.27)	4.55 (1)		*.03*	
	SHY^i^	3.47 (1.91)	3.42 (1.78)	4.19 (2.60)	4.11 (2.43)	0.01 (1)		.90	
	Total	55.40 (10.63)	55.00 (10.57)	55.43 (14.59)	53.28 (14.79)	1.04 (1)		.31	

^a^MOON: *The Secret Trail of Moon*.

^b^GASA: Game Addiction Scale for Adolescents.

^c^SDSC: Sleep Disturbance Scale for Children.

^d^DIMS: disorders of initiating and maintaining sleep.

^e^SBD: sleep breathing disorders.

^f^DA: disorders of arousal nightmares.

^g^SWTD: sleep-wake transition disorders.

^h^DOES: disorders of excessive somnolence.

^i^SHY: sleep hyperhidrosis.

#### Adherence and Acceptability

Additional information was collected using an unvalidated scale (previously applied in our usability study [47] to obtain the patients’ opinion of the video game). Of the 33 participants who played MOON, 21 (64%) played video games on a habitual basis and 20 (61%) had tried VR before. In total, 82% (27/33) of the participants liked the experience; 79% (26/33) of the participants would recommend it to other people with ADHD.

Additional information was collected on fatigue, boredom, or dizziness (in the case of VR). In total, 24% (8/33) of the participants experienced fatigue (not surprising in cognitive training), 13% (4/33) found it boring, and 7% (2/33) were dizzy on ≥1 occasions. The overall experience was considered positive, as reported by the parents. Some parents mentioned perceiving improvement in thinking before acting, responsibility, autonomy, and organization; others did not perceive changes. Some parents considered that we should have held more weekly sessions.

Most of the participants and their parents preferred face-to-face sessions to web-based sessions. Bugs (video game errors) were considered especially frustrating in the web-based sessions by both children and parents, especially in the interface or in games such as chess. The levels were considered well balanced, except for Kuburi (too demanding and frustrating). Overall, the video game music was liked by the participants; some patients said it helped them to concentrate.

## Discussion

### Principal Findings

In this prospective, single-center, randomized, unblinded pre-post evaluation study, our primary hypothesis of a 3-point decrease in the global SDQ score for emotional regulation after 3 months of MOON cognitive training in clinically stable, medicated children and adolescents with ADHD was not confirmed. However, we observed improvement trends in specific areas among patients who were more engaged with the MOON treatment. After conducting an ANCOVA with the number of sessions as a covariate, we found values approaching significance for the main hypothesis, with a 2-point drop in the global SDQ. This suggests that motivation may have played a key role in the improvement of emotional regulation in the participants. Given that motivation is a crucial factor in individuals with ADHD, it likely influenced these outcomes [21,24,31].

Considering the number of sessions as a covariate, significant differences in emotional symptoms and internalizing problems were found in favor of the experimental group. No behavioral improvement trends were observed. While externalizing difficulties are related to temperamental negative affect and anger dysregulation (bottom-up processes), internalizing difficulties suggest an overregulation of negative emotion through maladaptive cognitive strategies such as blaming and ruminating (top-down processes, more closely linked to executive functions) [48-50]. Participants with externalizing problems may have found the video game lacking in external rewards (eg, prizes), unlike those participants with greater intrinsic motivation (eg, self-improvement and sense of progress). This clinical trial had significantly more sessions than the previous one (20 vs 12) [29], increasing the need for novelty, change, and reinforcement. However, as participants noted in their feedback, the game did not seem to meet these reward-based needs effectively.

Our results also showed improvements in material organization, working memory, and inhibition in the 16-session MOON group. The improvement in working memory is in accordance with other investigations whose improvement in working memory occurred especially in those individuals who had a higher level of motivation and voluntary commitment to training [21,51] as well as the organization [52]. The ITT group also had an improvement close to significance. However, in the Corsi cubes (objective task of working memory), no significant differences were found; the MOON group had higher memory span than the control group, but this difference was very small. These results are consistent with other randomized controlled trials that showed significant improvements in ADHD symptoms and working memory but no improvements in other cognitive functions tested, such as planning or self-monitoring [53]. Regarding cognitive flexibility, significant differences were found in favor of the MOON group in set shifting on the CTMT-2 test, but it was not significant on the parent-reported flexibility subscale on the BRIEF-2 questionnaire.

Findings may point at a more powerful experience when using the VR experience. At least theoretically, VR may be more effective in tricking the brain than regular video games [28-30,47] suggesting a heightened sense of presence in the virtual environment and reduced perception of the real world, leading to fewer distractions [22]. In addition our findings may also just be a matter of age and brain maturity, perhaps related to the age of the VR users (aged >12 years years). Another putative explanation is that those who played on the computer experienced a more bugged and, consequently, more frustrating version of the game.

Furthermore, the use of MOON was well tolerated, as no clinically significant side effects were reported. There were no adverse events to report to the Spanish Agency of Medicines and Health Products. The VR intervention reported a mean dizziness score of 0.31 on the UKU scale, where 0 indicates no side effects and 1 indicates mild side effects, which did not interfere with patient performance. No significant differences were found in the GASA scale on video game addiction. In ADHD, this variable is especially important as there is a higher risk of addiction [54]. Serious video games versus commercial games allow controlling design variables about addiction [28]. Here, we found significant differences related to sleep in the subscale disorders of excessive somnolence in favor of the MOON group. As for the MOON group that played through VR, we also found that some of them remembered more of their dreams after the sessions, as assessed with the UKU scale. This phenomenon (although neither significant nor interfering) was consistent with the previous clinical trial [29]. While we do not consider this a concerning side effect, it is worth exploring the potential impact of VR on sleep.

This clinical trial has important limitations, particularly the smaller-than-planned sample size (n=152) that affected its statistical power [30]. Trends of improvement were observed in various areas, but a larger sample may have yielded statistically significant results. In addition, this investigation, such as the previous one, was not blinded [29]. The effect sizes are small, and we must interpret these results cautiously. The sessions were decreasing with the intervention with MOON on the web, and this suggests that the game was insufficiently developed, as mentioned by the users themselves. The reward system in the video game was only partially developed, which may have impacted motivation and caused the game to become repetitive. In addition, usability study would have been necessary before the clinical trial (as in the previous study [47] to refine the gameplay and correct bugs). Another limitation of the study was that hypothesis 4 could not be tested, and the corresponding analyses could not be performed, as academic grades were not collected consistently (only half of the parents provided this information).

Nonetheless, our results provide additional, albeit partial, support for incorporating serious video games into the multimodal treatment approach for ADHD. After conducting our second clinical trial, we considered that video games do not have to be for everyone. Heterogeneity in ADHD is substantial. While some patients found improvements related to cognitive training with MOON, others did not. Other training methods exist. To optimize the treatment of patients with ADHD, it is crucial to focus on individual interests and their strengths and difficulties to personalize the treatment to what the person needs. Video games can be another addition to complement multimodal treatment along with other interventions such as music training, physical exercise, regular interaction with nature, or learning a new language accompanied by a healthy peer support network and appropriate parenting. For example, to improve externalizing symptomatology, behavioral psychological therapy intervention strategies may be more effective, especially early in the preschool years [17]. Combining training with pharmacological treatment seems to enhance the benefits more than pharmacological treatment alone [23].

### Conclusions

Serious video games combined with multimodal treatment can improve symptoms associated with ADHD. In this study, significant differences in inhibition, working memory, and material organization were observed in participants who were more engaged in the MOON treatment.

## Appendix

Multimedia Appendix 1 CONSORT (Consolidated Standards of Reporting Trials) 2010 checklist of information to include when reporting a randomized trial.

Multimedia Appendix 2 Secondary outcomes related to hypotheses 4: Patients with attention-deficit/hyperactivity disorder using The Secret Trail of Moon improve in academic performance compared with the control group.

Multimedia Appendix 3 Estimated marginal means pre-post measured with the Strengths and Difficulties Questionnaire (SDQ) for group 1: The Secret Trail of Moon (green solid line) and group 2: control (purple dashed line). The drop in score indicates symptom improvement. The prosocial scale of the SDQ is an exception, where improvement is indicated by an increase in the score.

Multimedia Appendix 4 Estimated marginal means measured with the Strengths and Difficulties Questionnaire (SDQ) at 3 time points (pre, mid, and post) for group 1: The Secret Trail of Moon (green solid line) and group 2: control (purple dashed line). The drop in score indicates symptom improvement. The prosocial scale of the SDQ is an exception, where improvement is indicated by an increase in the score.

Multimedia Appendix 5 Number of web-based sessions completed by The Secret Trail of Moon group.

Multimedia Appendix 6 Estimated marginal means of pre-post attention-deficit/hyperactivity disorder symptoms measured with the Swanson, Nolan, and Pelham Rating Scale and Conners Abbreviated Symptom Questionnaire for group 1: The Secret Trail of Moon (green solid line) and group 2: control (purple dashed line). The drop in both scores indicates symptom improvement.

Multimedia Appendix 7 Estimated marginal means of pre-post cognitive functioning measured with T scores of the Behavior Rating Inventory Executive Function, version 2, questionnaire for parents. Pre-post comparisons for the 2 groups: The Secret Trail of Moon (green solid line) and group 2: control (purple dashed line). Decreasing scores indicate symptom improvement.

Multimedia Appendix 8 Estimated marginal means of pre-post cognitive functioning measured with T scores of neuropsychological tests (Corsi cubes; and Comprehensive Trail-Making Test, Second Edition [CTMT-2]) for patients with attention-deficit/hyperactivity disorder. Pre-post comparisons for the 2 groups: The Secret Trail of Moon (green solid line) and group 2: control (purple dashed line). Decreasing scores indicate symptom improvement, except in Corsi cubes and CTMT-2 whose improvement is a rise in score.

Multimedia Appendix 9 Total means of relevant symptoms and side effects assessed with the Udvalg für Kliniske Undersogelser questionnaire (all 1).

Multimedia Appendix 10 List of side effects measured with the Udvalg für Kliniske Undersogelser test during 10 face-to-face sessions.

## Abbreviations

ADHD: attention-deficit/hyperactivity disorder
ANCOVA: analysis of covariance
BRIEF-2: Behavior Rating Inventory Executive Function, version 2
CGI: Clinical Global Impression
CPRS: Conners Abbreviated Symptom Questionnaire
CPT-3: Conners’ Continuous Performance Test Third Edition
eCRF: electronic case report form
GASA: Game Addiction Scale for Adolescents
ITT: intention-to-treat
MOON: The Secret Trail of Moon
PP: per-protocol
REDCap: Research Electronic Data Capture
SDQ: Strengths and Difficulties Questionnaire
SNAP-IV: Swanson, Nolan, and Pelham Rating Scale
UKU: Udvalg für Kliniske Undersogelser
VR: virtual reality
