# Supplementary material for: Effectiveness of a Virtual Reality Serious Video Game (The Secret Trail of Moon) for Emotional Regulation in Children With Attention-Deficit/Hyperactivity Disorder: Randomized Clinical Trial
Source: JMIR Serious Games. 2025 Jan 8;13:e59124. doi: 10.2196/59124 (PMC11754979; doi:10.2196/59124)
Supplement: Multimedia Appendix 2 [file games_v13i1e59124_app2.docx]

| Academic performance | |  | | | |  | | | |  |  |
| --- | --- | --- | --- | --- | --- | --- | --- | --- | --- | --- | --- |
|  | | MOON | | | | Control | | | |  |  |
|  | | n | Pre  Mean (SD) | n | Post  Mean (SD) | n | Pre  Mean (SD) | n | Post  Mean (SD) | F | *p* value |
|  | Mathematics | 20 | 5.5 (2.06) | 18 | 5.7 (1.99) | 18 | 7.1 (1.45) | 16 | 5.5 (1.75) | .57 | .45 |
|  | Spanish Language | 20 | 5.5 (2.01) | 18 | 5.8 (1.58) | 18 | 6.5 (1.19) | 16 | 6.0 (1.18) | .20 | .65 |
|  | Foreign Language (English) | 20 | 6.0 (2.35) | 18 | 5.3 (2.3) | 18 | 7.0 (1.49) | 16 | 6.0 (1.76) | .07 | .79 |
|  | Physical Education | 19 | 7.4 (1.42) | 15 | 7.7 (1.16) | 18 | 7.7 (1.22) | 16 | 7.7 (1.73) | .62 | .43 |
|  | History | 19 | 5.8 (2.05) | 18 | 5.5 (2.33) | 18 | 7.2 (1.39) | 14 | 6.8 (1.51) | .49 | .49 |
|  | Art | 15 | 7.9 (1.38) | 10 | 7.8 (1.13) | 17 | 8.0 (2.12) | 10 | 8.5 (1.50) | .04 | .83 |
|  | Music | 16 | 6.8 (1.25) | 12 | 7.0 (1.41) | 16 | 8.1 (1.51) | 13 | 7.6 (1.93) | .09 | .76 |
|  | Technology | 11 | 6.3 (2.20) | 9 | 6.5 (1.33) | 6 | 8.3 (1.03) | 10 | 6.10(1.72) | 1.84 | .20 |
|  | Religious Studies | 11 | 6.9 (1.51) | 8 | 7.0 (1.33) | 11 | 7.45 (1.44) | 8 | 7.75 (1.75) | 1.31 | .27 |
