# Supplementary figures and images for: Effectiveness of a Virtual Reality Serious Video Game (The Secret Trail of Moon) for Emotional Regulation in Children With Attention-Deficit/Hyperactivity Disorder: Randomized Clinical Trial
Source: JMIR Serious Games. 2025 Jan 8;13:e59124. doi: 10.2196/59124 (PMC11754979; doi:10.2196/59124)

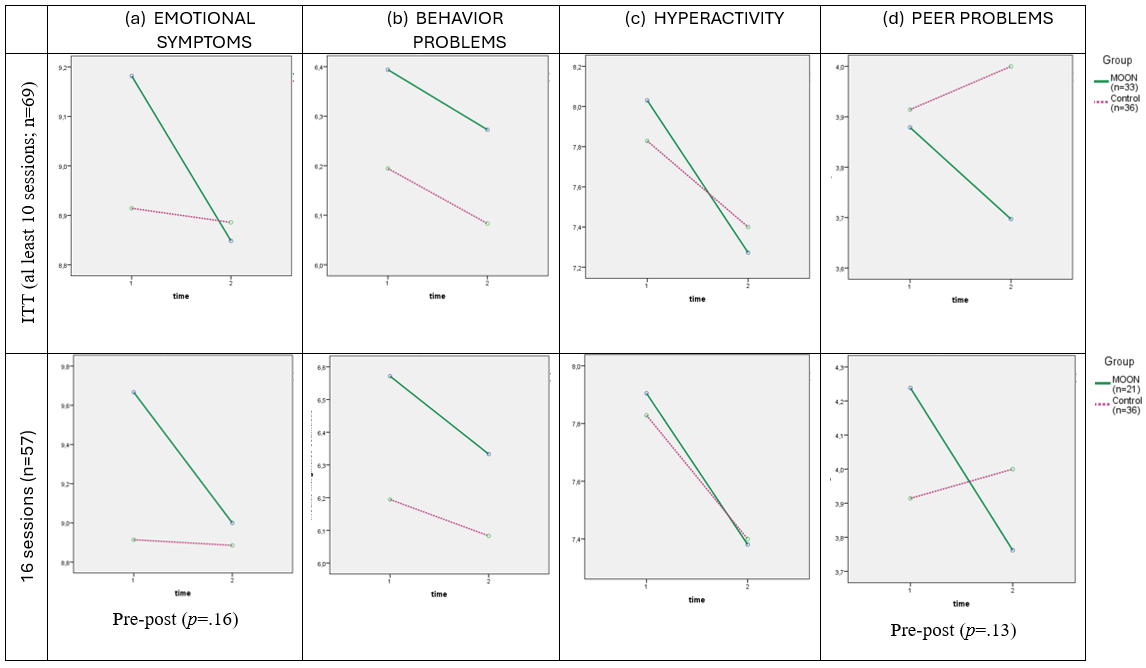


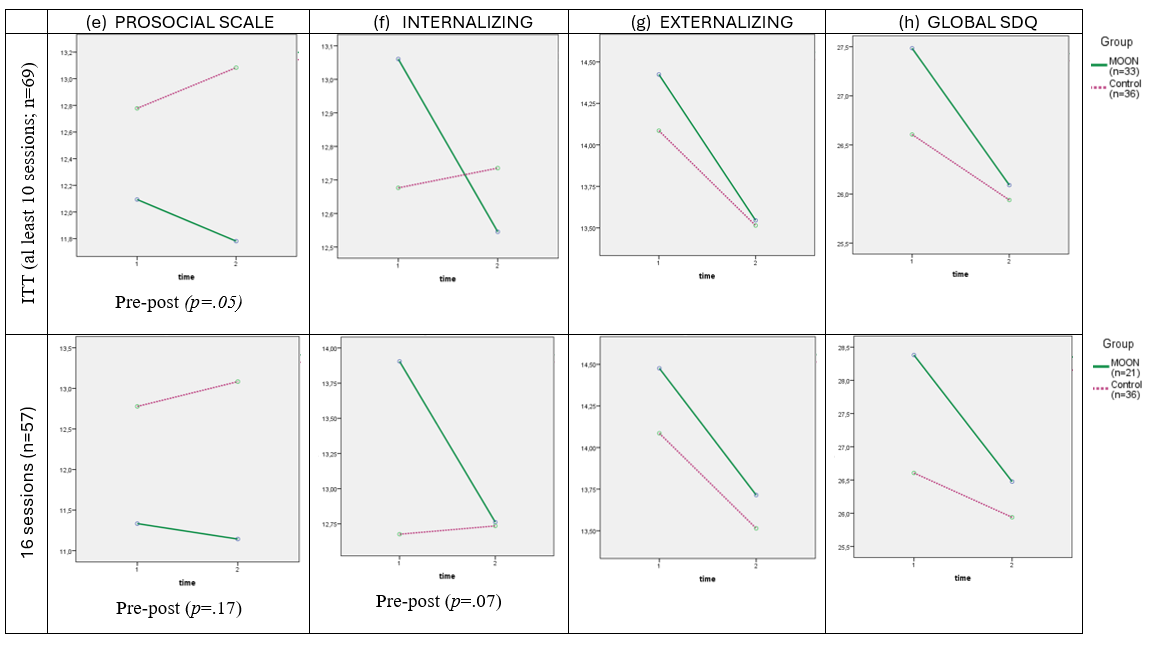

Supplement: Multimedia Appendix 3 [file games_v13i1e59124_app3.docx]

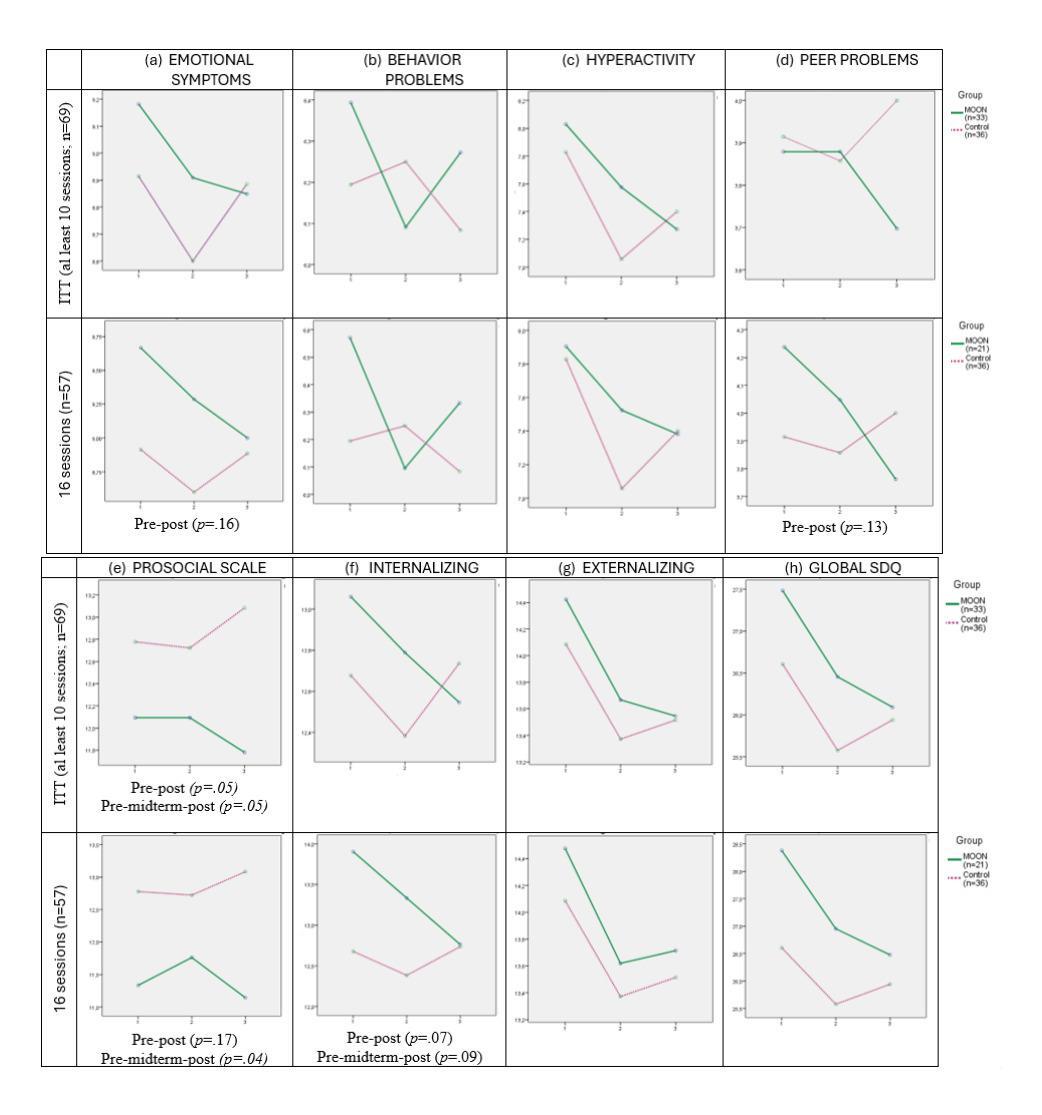

Supplement: Multimedia Appendix 4 [file games_v13i1e59124_app4.docx]

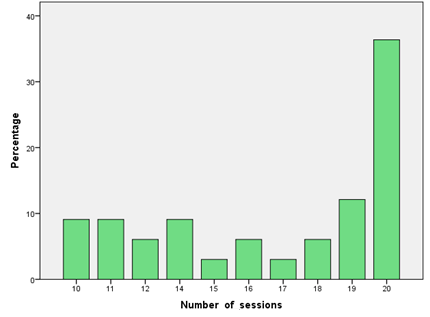

Supplement: Multimedia Appendix 5 [file games_v13i1e59124_app5.png]

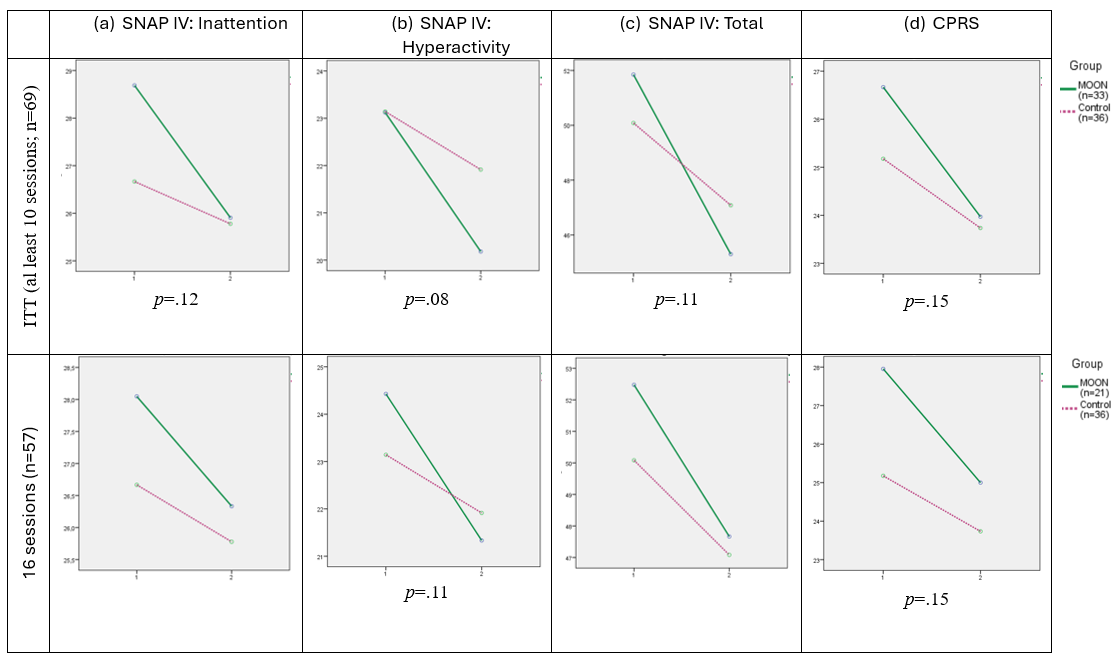

Supplement: Multimedia Appendix 6 [file games_v13i1e59124_app6.docx]

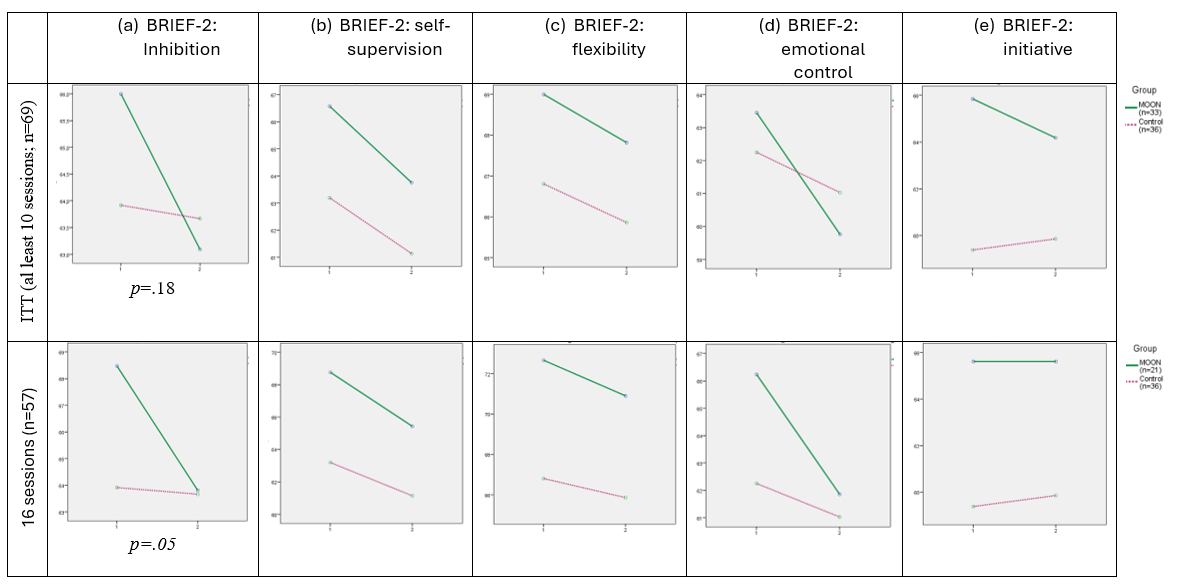


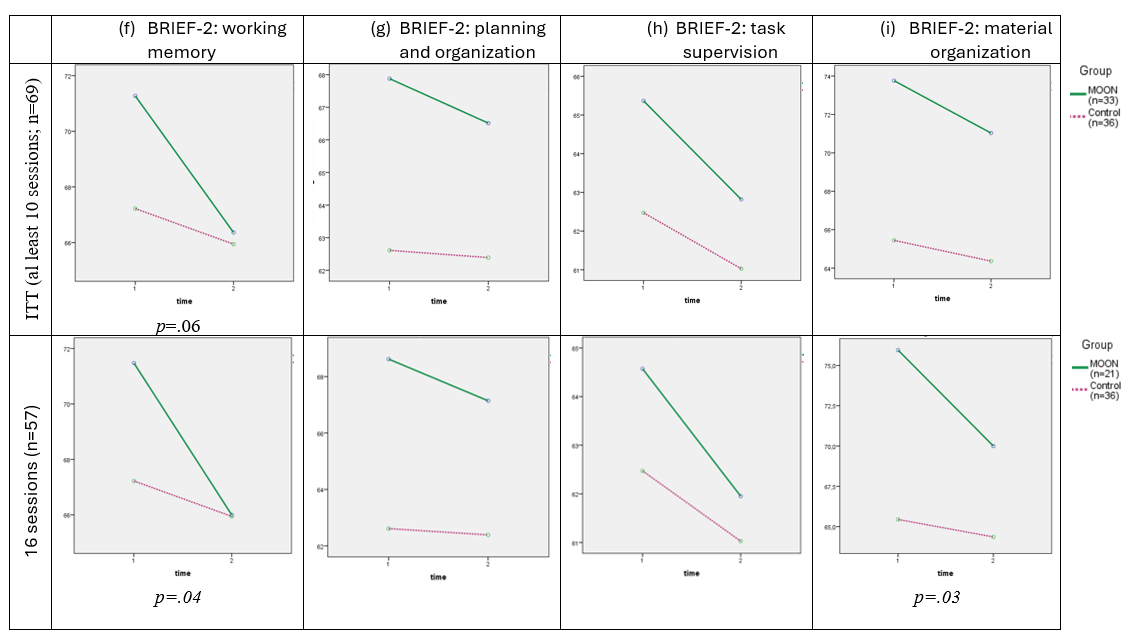


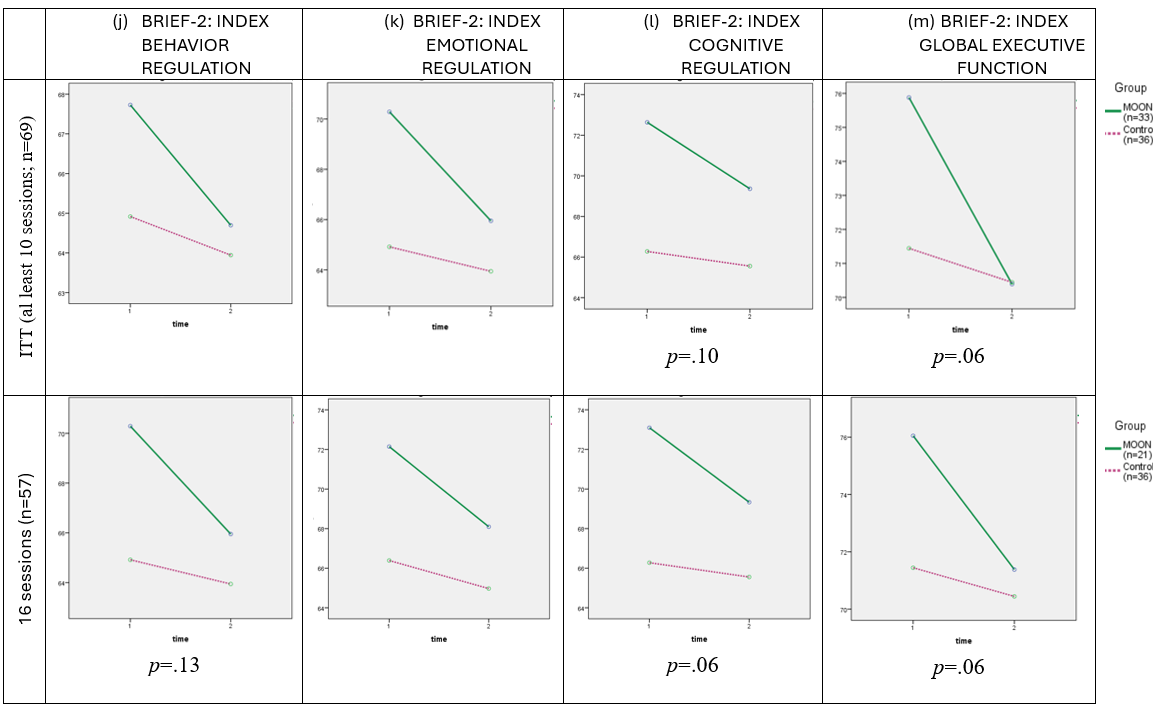

Supplement: Multimedia Appendix 7 [file games_v13i1e59124_app7.docx]

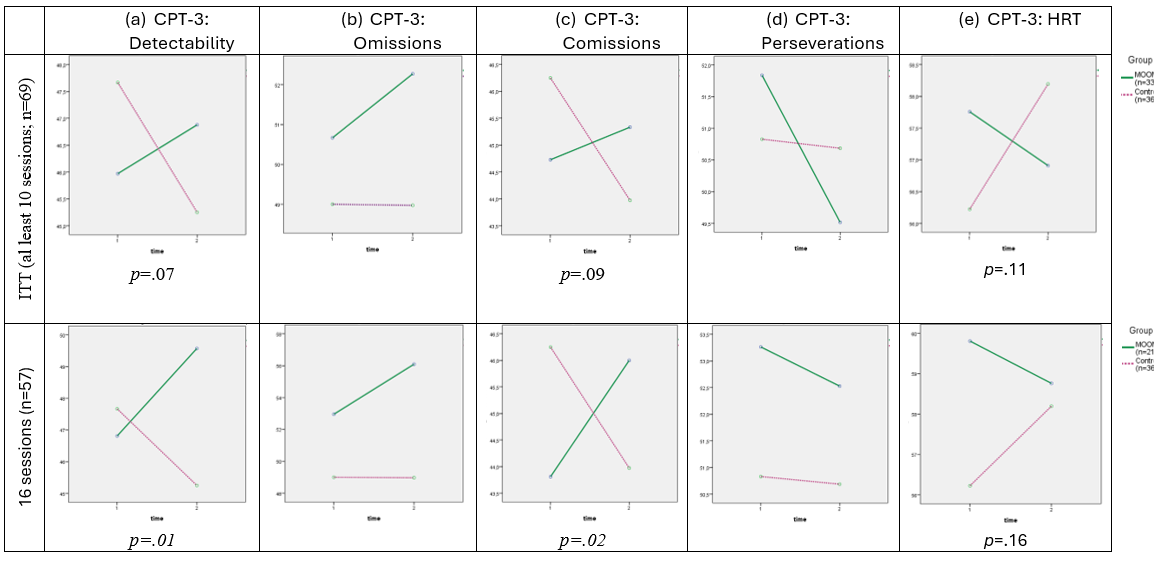


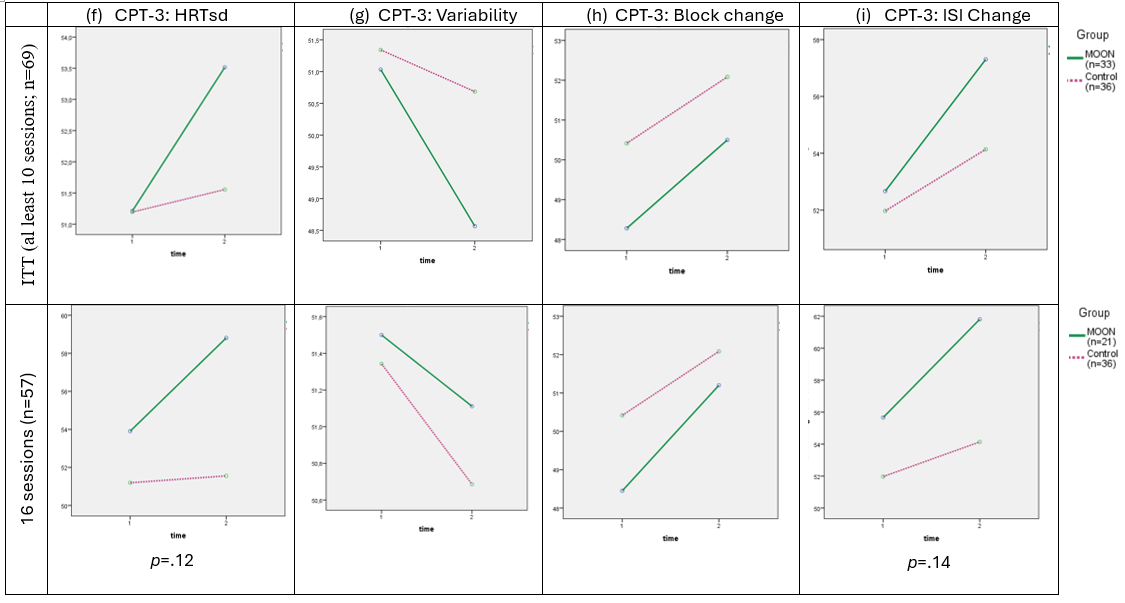


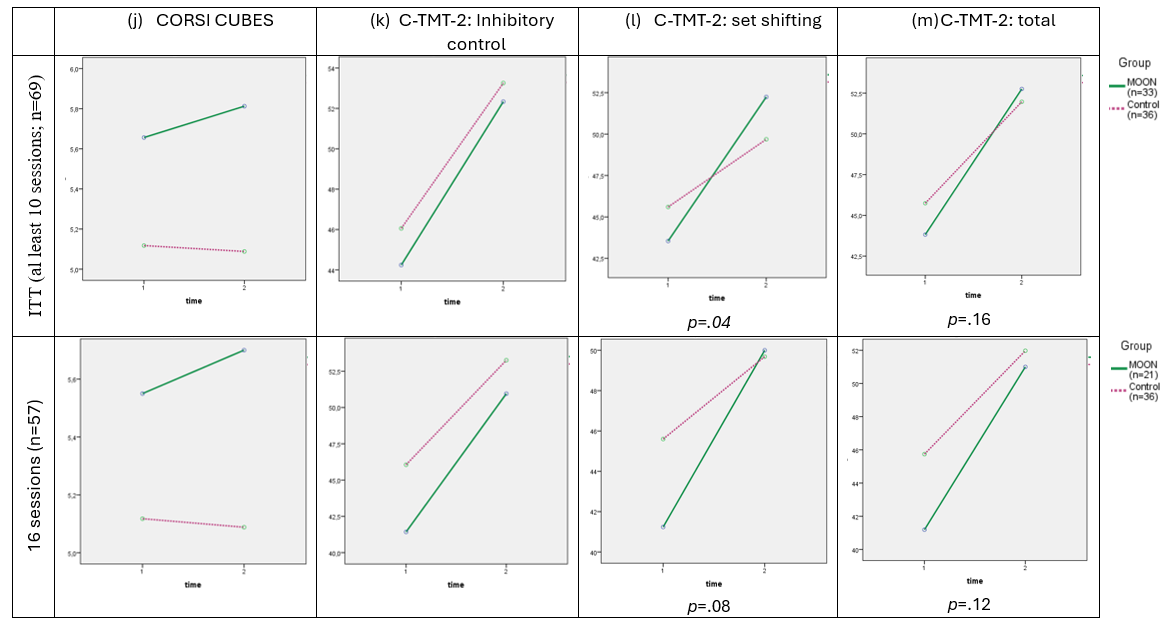

Supplement: Multimedia Appendix 8 [file games_v13i1e59124_app8.docx]

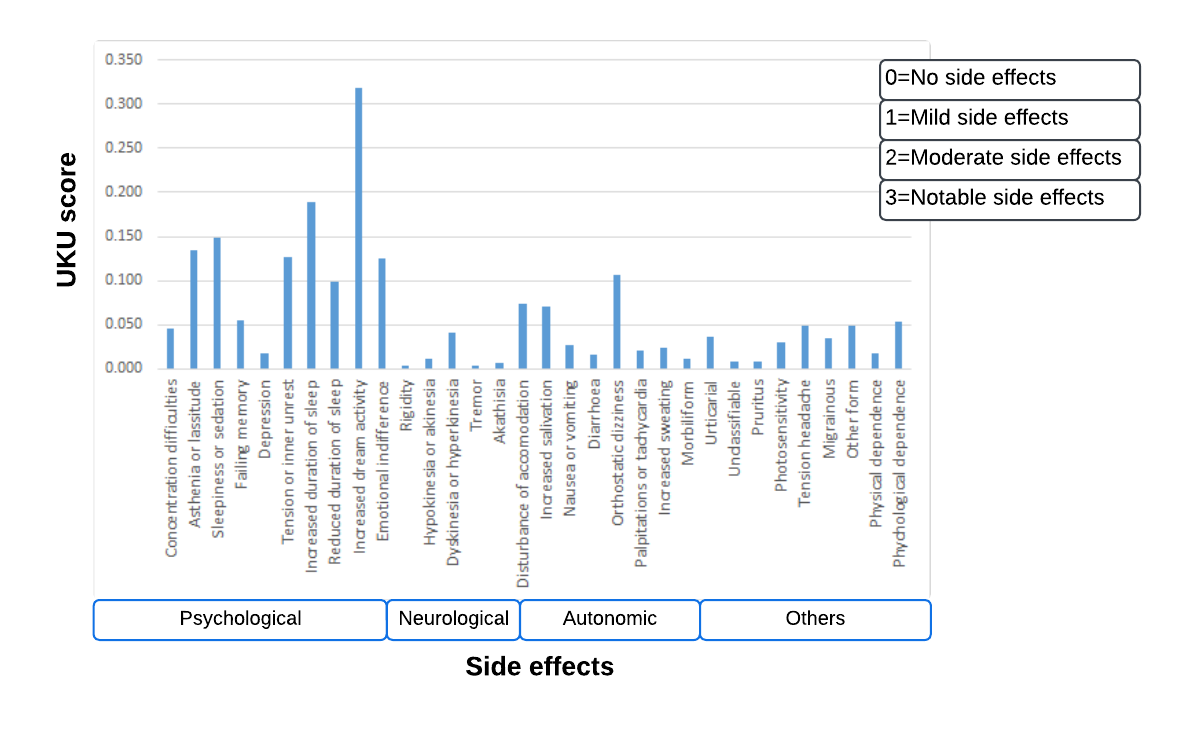

Supplement: Multimedia Appendix 9 [file games_v13i1e59124_app9.png]
