# Supplementary material for: Effectiveness of a Virtual Reality Serious Video Game (The Secret Trail of Moon) for Emotional Regulation in Children With Attention-Deficit/Hyperactivity Disorder: Randomized Clinical Trial
Source: JMIR Serious Games. 2025 Jan 8;13:e59124. doi: 10.2196/59124 (PMC11754979; doi:10.2196/59124)
Supplement: Multimedia Appendix 10 [file games_v13i1e59124_app10.docx]

|  |  |  |  |  |  |  | 0= No side effects | | |  |  |  |  |
| --- | --- | --- | --- | --- | --- | --- | --- | --- | --- | --- | --- | --- | --- |
|  |  |  |  |  |  |  | 1= Mild side effects | | |  |  |  |  |
|  |  |  |  |  |  |  | 2= Moderate side effects | | |  |  |  |  |
|  |  |  |  |  |  |  | 3= Notable side effects | | |  |  |  |  |
|  |  |  |  |  |  |  |  |  |  |  |  |  |  |
|  |  | Session 1 | Session 2 | Session 3 | Session 4 | Session 5 | Session 6 | Session 7 | Session 8 | Session 9 | Session 10 |  |  |
| Psychological | Concentration Difficulties | 0,11 | 0,15 | 0,04 | 0,07 | 0 | 0 | 0 | 0 | 0,04 | 0,04 | 0,045 |  |
| Psychological | **Asthenia/Lassitude** | 0,26 | 0,11 | 0,26 | 0,3 | 0,15 | 0,04 | 0,04 | 0,04 | 0,11 | 0,04 | **0,135** |  |
| Psychological | **Sleepiness/Sedation** | 0,3 | 0,11 | 0,3 | 0,15 | 0,26 | 0 | 0,11 | 0,07 | 0,11 | 0,07 | **0,148** |  |
| Psychological | Failing Memory | 0,07 | 0,07 | 0,04 | 0,07 | 0,11 | 0 | 0,04 | 0 | 0 | 0,15 | 0,055 |  |
| Psychological | Depression | 0 | 0,07 | 0,07 | 0 | 0,04 | 0 | 0 | 0 | 0 | 0 | 0,018 |  |
| Psychological | Tension/Inner Unrest | 0,22 | 0,19 | 0,22 | 0,19 | 0,19 | 0,04 | 0,04 | 0,07 | 0,04 | 0,07 | **0,127** |  |
| Psychological | **Increased Duration of Sleep** | 0,22 | 0,33 | 0,44 | 0,04 | 0,22 | 0,19 | 0,15 | 0,19 | 0,11 | 0 | **0,189** |  |
| Psychological | **Reduced Duration of Sleep** | 0,04 | 0,11 | 0,19 | 0,15 | 0,11 | 0,07 | 0,07 | 0,11 | 0,07 | 0,07 | 0,099 |  |
| Psychological | **Increased Dream Activity** | 0,15 | 0,3 | 0,52 | 0,22 | 0,22 | 0,22 | 0,41 | 0,22 | 0,41 | 0,52 | **0,319** | Possible and Probable |
| Psychological | **Emotional Indifference** | 0,33 | 0,26 | 0,26 | 0,11 | 0 | 0,22 | 0 | 0,07 | 0 | 0 | **0,125** |  |
| Neurological | Dystonia | 0 | 0 | 0 | 0 | 0 | 0 | 0 | 0 | 0 | 0 | 0,000 |  |
| Neurological | Rigidity | 0 | 0,04 | 0 | 0 | 0 | 0 | 0 | 0 | 0 | 0 | 0,004 |  |
| Neurological | Hypokinesia/Akinesia | 0,11 | 0 | 0 | 0 | 0 | 0 | 0 | 0 | 0 | 0 | 0,011 |  |
| Neurological | Dyskinesia/Hyperkinesia | 0,33 | 0,04 | 0 | 0 | 0 | 0,04 | 0 | 0 | 0 | 0 | 0,041 |  |
| Neurological | Tremor | 0 | 0 | 0 | 0,04 | 0 | 0 | 0 | 0 | 0 | 0 | 0,004 |  |
| Neurological | Akathisia | 0 | 0,07 | 0 | 0 | 0 | 0 | 0 | 0 | 0 | 0 | 0,007 |  |
| Neurological | Epileptic Seizures | 0 | 0 | 0 | 0 | 0 | 0 | 0 | 0 | 0 | 0 | 0,000 |  |
| Neurological | Paraesthesias | 0 | 0 | 0 | 0 | 0 | 0 | 0 | 0 | 0 | 0 | 0,000 |  |
| Autonomic | Disturbance of Accomodation | 0,26 | 0,11 | 0,07 | 0,11 | 0,07 | 0,04 | 0,04 | 0 | 0 | 0,04 | 0,074 | Probable |
| Autonomic | Increased Salivation | 0,07 | 0,11 | 0,19 | 0,07 | 0,07 | 0,04 | 0,11 | 0,04 | 0 | 0 | 0,070 |  |
| Autonomic | Reduced Salivation | 0 | 0 | 0 | 0 | 0 | 0 | 0 | 0 | 0 | 0 | 0,000 |  |
| Autonomic | Nausea/Vomiting | 0 | 0,11 | 0 | 0,11 | 0,04 | 0 | 0 | 0 | 0 | 0 | 0,026 |  |
| Autonomic | Diarrhoea | 0 | 0,04 | 0 | 0,11 | 0 | 0 | 0 | 0 | 0 | 0 | 0,015 |  |
| Autonomic | Constipation | 0 | 0 | 0 | 0 | 0 | 0 | 0 | 0 | 0 | 0 | 0,000 |  |
| Autonomic | Disturbance of Micturition | 0 | 0 | 0 | 0 | 0 | 0 | 0 | 0 | 0 | 0 | 0,000 |  |
| Autonomic | Polyuria/Poydipsia | 0 | 0 | 0 | 0 | 0 | 0 | 0 | 0 | 0 | 0 | 0,000 |  |
| Autonomic | **Orthostatic Dizziness** | 0,07 | 0,11 | 0,22 | 0,26 | 0,07 | 0 | 0,11 | 0,15 | 0,04 | 0,04 | **0,107** | Probable |
| Autonomic | Palpitations/Tachycardia | 0 | 0,04 | 0,04 | 0,04 | 0,04 | 0,04 | 0 | 0 | 0 | 0 | 0,020 |  |
| Autonomic | Increased Sweating | 0 | 0,07 | 0 | 0,04 | 0 | 0,04 | 0 | 0,04 | 0 | 0,04 | 0,023 |  |
| Others | Morbiliform | 0,07 | 0 | 0 | 0,04 | 0 | 0 | 0 | 0 | 0 | 0 | 0,011 |  |
| Others | Petechial | 0 | 0 | 0 | 0 | 0 | 0 | 0 | 0 | 0 | 0 | 0,000 |  |
| Others | Urticarial | 0 | 0,07 | 0 | 0,11 | 0,11 | 0,07 | 0 | 0 | 0 | 0 | 0,036 |  |
| Others | Psoriatic | 0 | 0 | 0 | 0 | 0 | 0 | 0 | 0 | 0 | 0 | 0,000 |  |
| Others | Unclassifiable | 0,04 | 0 | 0,04 | 0 | 0 | 0 | 0 | 0 | 0 | 0 | 0,008 |  |
| Others | Pruritus | 0,04 | 0 | 0 | 0 | 0,04 | 0 | 0 | 0 | 0 | 0 | 0,008 |  |
| Others | Photosensitivity | 0,07 | 0,04 | 0,04 | 0 | 0,04 | 0 | 0,07 | 0,04 | 0 | 0 | 0,030 |  |
| Others | Increased pigmentation | 0 | 0 | 0 | 0 | 0 | 0 | 0 | 0 | 0 | 0 | 0,000 |  |
| Others | Weight gain | 0 | 0 | 0 | 0 | 0 | 0 | 0 | 0 | 0 | 0 | 0,000 |  |
| Others | Weight loss | 0 | 0 | 0 | 0 | 0 | 0 | 0 | 0 | 0 | 0 | 0,000 |  |
| Others | Menorrhagia | 0 | 0 | 0 | 0 | 0 | 0 | 0 | 0 | 0 | 0 | 0,000 |  |
| Others | Amenorrhea | 0 | 0 | 0 | 0 | 0 | 0 | 0 | 0 | 0 | 0 | 0,000 |  |
| Others | Galactorrhea | 0 | 0 | 0 | 0 | 0 | 0 | 0 | 0 | 0 | 0 | 0,000 |  |
| Others | Gynaecomastia | 0 | 0 | 0 | 0 | 0 | 0 | 0 | 0 | 0 | 0 | 0,000 |  |
| Others | Increased sexual desire | 0 | 0 | 0 | 0 | 0 | 0 | 0 | 0 | 0 | 0 | 0,000 |  |
| Others | Decreased sexual desire | 0 | 0 | 0 | 0 | 0 | 0 | 0 | 0 | 0 | 0 | 0,000 |  |
| Others | Erectil dysfunction | 0 | 0 | 0 | 0 | 0 | 0 | 0 | 0 | 0 | 0 | 0,000 |  |
| Others | Ejaculatory dysfunction | 0 | 0 | 0 | 0 | 0 | 0 | 0 | 0 | 0 | 0 | 0,000 |  |
| Others | Orgasmic dysfunction | 0 | 0 | 0 | 0 | 0 | 0 | 0 | 0 | 0 | 0 | 0,000 |  |
| Others | Vaginal lubrication | 0 | 0 | 0 | 0 | 0 | 0 | 0 | 0 | 0 | 0 | 0,000 |  |
| Others | Tension Headache | 0 | 0,11 | 0,07 | 0,07 | 0,15 | 0 | 0 | 0 | 0,04 | 0,04 | 0,048 | Probable |
| Others | Migrainous | 0,11 | 0 | 0 | 0 | 0,19 | 0 | 0 | 0 | 0,04 | 0 | 0,034 |  |
| Others | Other form | 0 | 0,04 | 0,19 | 0,07 | 0,04 | 0 | 0,07 | 0,07 | 0 | 0 | 0,048 | Probable |
| Others | Physical dependence | 0 | 0,07 | 0 | 0 | 0 | 0 | 0,07 | 0 | 0 | 0,04 | 0,018 |  |
| Others | Phychological dependence | 0,19 | 0,04 | 0,04 | 0,07 | 0,11 | 0 | 0 | 0 | 0,04 | 0,04 | 0,053 |  |
|  |  | 0,057 | 0,052 | 0,060 | 0,045 | 0,042 | 0,019 | 0,025 | 0,021 | 0,019 | 0,022 |  |  |
